# Relative contributions of pre-pandemic factors and intra-pandemic activities to differential COVID-19 risk among migrant and non-migrant populations in the Netherlands: lessons for future pandemic preparedness

**DOI:** 10.1186/s12939-023-01936-0

**Published:** 2023-07-04

**Authors:** Felix P. Chilunga, Sophie Campman, Henrike Galenkamp, Anders Boyd, Renee Bolijn, Tjalling Leenstra, Charles Agyemang, Ellen Uiters, Maria Prins, Karien Stronks

**Affiliations:** 1 Department of Public and Occupational Health, Amsterdam UMC, Amsterdam Public Health Research Institute, University of Amsterdam, Amsterdam, The Netherlands; 2 Department of Infectious Diseases, Public Health Service Amsterdam (GGD Amsterdam), Amsterdam, The Netherlands; 3 Department of Infectious Diseases, Amsterdam Infection, and Immunity (AII), Amsterdam UMC, University of Amsterdam, Amsterdam, The Netherlands; 4 Stichting HIV Monitoring, Amsterdam, The Netherlands; 5 Centre for Infectious Disease Control, National Coordination Centre for Communicable Disease Control, National Institute for Public Health, and the Environment, Bilthoven, The Netherlands; 6 National Institute for Public Health and the Environment, Bilthoven, The Netherlands; 7 Centre for Urban Mental Health, University of Amsterdam, Amsterdam, The Netherlands

**Keywords:** Migrants and transients, Pandemic preparedness, COVID-19, SARS-CoV-2, Risk factors.

## Abstract

**Background:**

Although risk factors for differences in SARS-CoV-2 infections between migrant and non-migrant populations in high income countries have been identified, their relative contributions to these SARS-CoV-2 infections, which could aid in the preparation for future viral pandemics, remain unknown. We investigated the relative contributions of pre-pandemic factors and intra-pandemic activities to differential SARS-CoV-2 infections in the Netherlands by migration background (Dutch, African Surinamese, South-Asian Surinamese, Ghanaians, Turkish, and Moroccan origin).

**Methods:**

We utilized pre-pandemic (2011–2015) and intra-pandemic (2020–2021) data from the HELIUS cohort, linked to SARS-CoV-2 PCR test results from Public Health Service of Amsterdam (GGD Amsterdam). Pre-pandemic factors included socio-demographic, medical, and lifestyle factors. Intra-pandemic activities included COVID-19 risk aggravating and mitigating activities such as physical distancing, use of face masks, and other similar activities. We calculated prevalence ratios (PRs) in the HELIUS population that was merged with GGD Amsterdam PCR test data using robust Poisson regression (SARS-CoV-2 PCR test result as outcome, migration background as predictor). We then obtained the distribution of migrant and non-migrant populations in Amsterdam as of January 2021 from Statistics Netherlands. The migrant populations included people who have migrated themselves as well as their offspring. We used PRs and the population distributions to calculate population attributable fractions (PAFs) using the standard formula. We used age and sex adjusted models to introduce pre-pandemic factors and intra-pandemic activities, noting the relative changes in PAFs.

**Results:**

From 20,359 eligible HELIUS participants, 8,595 were linked to GGD Amsterdam PCR test data and included in the study. Pre-pandemic socio-demographic factors (especially education, occupation, and household size) resulted in the largest changes in PAFs when introduced in age and sex adjusted models (up to 45%), followed by pre-pandemic lifestyle factors (up to 23%, especially alcohol consumption). Intra-pandemic activities resulted in the least changes in PAFs when introduced in age and sex adjusted models (up to 16%).

**Conclusion:**

Interventions that target pre-pandemic socio-economic status and other drivers of health inequalities between migrant and non-migrant populations are urgently needed at present to better prevent infection disparities in future viral pandemics.

**Supplementary Information:**

The online version contains supplementary material available at 10.1186/s12939-023-01936-0.

## Background

One of the important topics during the Coronavirus disease 2019 (COVID-19) pandemic has been that of differences in SARS-CoV-2 infections between migrant and non-migrant populations in high income countries [1]. Specifically, migrant populations residing in high income countries were reported to have a disproportionately higher burden of SARS-CoV-2 infections than the non-migrant populations [2–5]. In response to these reports, researchers, policymakers, and other stakeholders took a variety of actions, such as identifying factors that could explain the differential risk,[6] as well as enacting COVID-19 prevention measures, including those aimed at the migrant populations [3, 7].

The spread and impact of COVID-19 among minority groups during the pandemic were influenced by various factors associated with migration status, rather than migration status itself. Specifically, factors such as being an essential worker (e.g., taxi drivers, social and health care workers), having underlying medical conditions, living in large households, and low socio-economic status, were identified as explanatory factors for the higher SARS-CoV-2 infection risk among migrant populations as compared to the non-migrant populations [6]. On the other hand, COVID-19 prevention methods included both direct and indirect measures, such as wearing face masks and physical distancing, as well as avoiding social gatherings and other potential sources of exposure [8]. Based on the identified factors and prevention methods, we propose that the risk of SARS-CoV-2 infection results from factors that were present before the pandemic (e.g., socio economic status, large households, underlying chronic illnesses, unhealthy lifestyles, immune status)(i.e., pre-pandemic factors) or from levels of adherence to COVID-19 prevention measures during the pandemic (i.e., intra-pandemic activities) [9, 10].

While previous studies have shown that pre-pandemic factors and intra-pandemic activities can explain differential SARS-CoV-2 infections between migrants and non-migrants,[3, 4, 6, 9, 10] no studies have yet quantified the relative contributions of these factors/activities to the variations in SARS-CoV-2 infections. Understanding the relative contributions of pre-pandemic factors and intra-pandemic activities is essential for preparing for future infectious disease pandemics now that the COVID-19 pandemic is largely under control. For instance, interventions on pre-pandemic factors could be implemented before the next infectious disease pandemic, or a response plan targeting intra-pandemic activities could be developed for these future pandemics.

We therefore assessed relative contributions of pre-pandemic factors (socio-demographic, medical, and lifestyle factors) and intra-pandemic activities (COVID-19 risk aggravating and mitigating activities) to differential SARS-CoV-2 infections in Amsterdam, the Netherlands, by migration background.

## Methods

### Study population and design

The baseline of the Healthy Life in an Urban Setting (HELIUS) cohort (2011–2015) and two of its COVID-19 sub-studies (2020–2021) were used in this cross-sectional analysis. While the primary HELIUS study gathered data on pre-pandemic factors, the two COVID-19 sub-studies focused on everyday activities during the pandemic itself. The HELIUS cohort data were also linked to SARS-CoV-2 polymerase chain reaction (PCR) results from the Public Health Service of Amsterdam (GGD Amsterdam). Below is a detailed explanation of the data sources and how they connect.

### The HELIUS cohort

The HELIUS cohort is a multi-ethnic population based prospective study initiated in 2011 in Amsterdam, the Netherlands, focusing on cardiovascular diseases, mental health, and infectious diseases. A full description of the cohort is provided elsewhere [11]. In brief, HELIUS includes a total of 24,782 persons of Dutch, Surinamese (classified into African, South-Asian, or ‘other’ based on self-report), Ghanaian, Turkish and Moroccan origin, aged between 18 and 70 years at inclusion. These countries of origin were selected because there represent the most common origin of migrant groups in the Netherlands. Participants were randomly sampled from the municipality register of Amsterdam by migration background. Migration background was based on the standard classification of Statistics Netherlands [12]. This standardized classification considers the country of birth of residents and their parents, thus includes immigrants’ descendants. Participants were considered of Dutch origin if they were born in the Netherlands and their parents were also born in the Netherlands, and as first and second-generation migrants if; (1) they were born abroad and had at least one parent born abroad (first generation) or (2) they were born in the Netherlands, but both parents were born abroad (second generation). The baseline study took place between 2011 and 2015, and participants completed a questionnaire as well as physical examination during which biological samples were obtained.

### The COVID-19 serological sub-study

The COVID-19 serological sub-study of the HELIUS cohort evaluated the incidence of SARS-CoV-2 infections by testing for antibodies and assessing factors that contributed to infection. The sub-study is described in detail elsewhere [3]. In summary, a total of 11,078 HELIUS participants were randomly selected by migration background and invited to take part in the sub-study. Subsequently, 2,497 participants took part in the sub-study (response rate 23%), which initially consisted of two visits. The first visit took place between June 24 and October 9, 2020 (early part of second wave of the pandemic in Netherlands), and the second between November 23, 2020, and June 4, 2021 (later part of second wave of the pandemic in Netherlands). Serum samples were obtained via venepuncture for SARS-CoV-2 antibody testing during both visits. Participants were also interviewed by trained interviewers who inquired about SARS-CoV-2 exposure, COVID-19 symptoms, and activities that increase the likelihood of contracting COVID-19 (e.g., going out for religious or recreational activities).

### The online COVID-19 sub-study

Between August 27, 2020, and September 29, 2020, the COVID-19 online sub-study of the HELIUS cohort was carried out (early part of second wave of the pandemic in Netherlands). The sub-study investigated how the spread of COVID-19 had affected people and how effective certain preventative measures had been. An in-depth account of the sub-study is presented elsewhere [13]. Briefly, 13,031 HELIUS participants that had email addresses were contacted and asked to fill out an online questionnaire. In the questionnaire, participants were asked about changes in their finances, lifestyle factors, mental health, use of non-COVID-19 health care, as well as how easy or hard it was for them to perform COVID-19 risk mitigating activities like handwashing and physical distancing. Thereafter, respondents provided a response from a series of Likert-scale options presented to them. The online survey was broken up into four parts so that respondents would only have to complete one part at a time, reducing the likelihood of non-responses due to survey length. A total of 1,105 respondents (out of the possible 4,450; response rate 25%) completed the section on measures taken to prevent the spread of COVID-19.

### Linkage to PCR test results

In the context of infectious disease control, the Public Health Service of Amsterdam (GGD Amsterdam; https://www.ggd.amsterdam.nl/) collected registry data on testing for SARS-CoV-2 in the Amsterdam-Amstelland region. Data protection and anonymization were prioritized throughout the process. The registry data were securely stored in a central database called CoronIT. To link the HELIUS data with the testing data from CoronIT, a deterministic linkage algorithm was used, which utilized a limited set of identifying information including last name, initials, sex, date of birth, and zip code. These measures were implemented to minimize the risk of re-identification and safeguard individual privacy. The registrations included data from the beginning of the Coronavirus pandemic until September 6, 2021. Only HELIUS participants who explicitly granted permission for linkage and were still alive on March 1, 2020, were linked to the CoronIT data. Participants who could not be linked to CoronIT data were assumed to have not been tested for SARS-CoV-2 using a PCR-based assay in the Amsterdam-Amstelland region. The linkage process was facilitated by a trusted third party, ZorgTTP, based in Houten, the Netherlands. ZorgTTP acted as an intermediary, ensuring the secure and confidential linkage of the HELIUS and CoronIT data. They developed a secure server for pseudonymization and linkage of the data. The HELIUS team and the GGD Amsterdam each entered the linkage data for eligible participants after which data was pseudonymised and linked. Furthermore, after the linkage process, all identifying information was excluded to ensure that only anonymized data was available for subsequent analyses.

### Ethical approval and informed consent

Ethical approval, in accordance with the ethical standards as laid down in the 1964 Declaration of Helsinki and its later amendments, for the HELIUS study was obtained from Academic Medical Center Ethical Review Board. All participants included in the study provided written informed consent for participation.

### Measurements

#### SARS-CoV-2 infection status

SARS-CoV-2 infection status was obtained from highly sensitive and specific PCR-based assay,[14] available from the CoronIT database at Amsterdam Public Health Service. The PCR tests were performed on nasopharyngeal swabs. Since participants could have multiple PCR tests, results were categorised as any positive test result vs. all negative results. PCR test results that were inconclusive were excluded.

### Pre-pandemic factors

Pre-pandemic factors were defined as COVID-19 risk factors that were present before the start of the pandemic. These factors were obtained from the main HELIUS cohort at baseline (2011–2015). They included sociodemographic factors (sex, education, occupation, household size), medical factors (health literacy and history of underlying health conditions), and lifestyle factors (alcohol consumption, tobacco smoking, fruit intake, physical activity). These factors were selected because they are part of the framework for understanding pathways underpinning inequalities in COVID-19 between migrants and non-migrants as proposed by Katikireddi et al. [10].

Sociodemographic factors: sex was categorised into male and female, highest educational level attained in the Netherlands or in the country of origin was categorised into never been to school or elementary school, lower vocational or secondary school, intermediate vocational or secondary school, and higher vocation school or university. Occupational level was classified according to the Dutch Standard Occupational Classification system which provides an extensive systematic list of all professions in the Dutch system [15]. These categories were elementary, lower, intermediary, higher occupations, and scientific occupations. Household size was categorised into one-person, two-person, three-person, four-person, and five-person plus households. Health literacy was measured using the validated set of brief screening questions (SBSQ) questionnaire, [15] and categorized into adequate or inadequate using cut-offs proposed by Chew et al. [16]

Medical factors: a list of relevant underlying health conditions was obtained from the National Institute for Public Health (RIVM, https://www.rivm.nl/en/coronavirus-covid-19/risk-groups). The following health conditions were available for inclusion in our study: hypertension, diabetes, obesity, kidney disease, asthma, and metabolic syndrome. Participants were further categorised as not having any of these conditions, having the condition but not receiving medication, and having the condition and receiving medication (i.e., anti-hypertensives, anti-asthma drugs, anti-diabetics).

Lifestyle factors: alcohol consumption was categorised as any or no consumption in the last year, tobacco smoking into never, past, and current smokers. Physical activity was measured by the short questionnaire to assess health-enhancing physical activity (SQUASH) and categorised into meeting and not meeting Dutch physical activity norm [16]. Fruit intake was measured as the number fruits a participant consumed in a week on average.

### Intra-pandemic COVID-19 risk aggravating activities

COVID-19 risk aggravating activities were defined as everyday activities that increased the risk of getting infected with SARS-CoV-2 during the pandemic. The activities included how frequent participants went out in the past week to shop for groceries, to visit family and friends, to visit recreational facilities, to visit religious places, to go to work, and to visit outdoor public places. Responses were recoded as yes or no. These data were obtained from the COVID-19 serological sub-study.

### Intra-pandemic COVID-19 risk mitigating activities

COVID-19 risk mitigating activities were defined as everyday activities that decreased SARS-CoV-2 infection risk as recommended by the Dutch government to control the pandemic. These activities included how participants found it easy or difficult to regularly wash hands with soap and water, to keep a physical distance to others, to wear a face mask in public, to cough and sneeze in the elbow, to stay home as much possible, and to not shake hands. Participants could answer with either very easy, easy, neutral, difficult, or very difficult. Those that found it easy to adhere to the activities were categorised together (easy and very easy) to compare them with those that found it neutral or difficult. These data were available from the COVID-19 online sub-study.

### Other measurements

Age was obtained from the main HELIUS cohort and reported as of 1 February 2021 (the mid-point of June 2020 to September 2021 HELIUS sub-study periods). It was categorized into 24–29, 30–34, 35–39, 40–44, 45–49, 50–54, 55–59, 60–64, 65–69, 70–75, 75–79 years. The distribution of migrant and non-migrant populations in Amsterdam as of 01 January 2021 was obtained from Statistics Netherlands (CBS, https://opendata.cbs.nl/statline/#/CBS/nl/dataset/84910NED/table?ts=1664356685421). The proportions of population groups were as follows: Dutch origin 43.83%, Ghanaian origin 1.49%, Turkish origin 5.08%, Moroccan origin 8.87% and Surinamese origin 7.3%. There was a lack of data on Surinamese sub-groups, but South-Asian Surinamese and African Surinamese were split into equal proportions of 3.65% based on the equal distribution of these sub-groups in our HELIUS cohort.

Vaccination against COVID-19, which was widely available during the second time point of the serological study was obtained as “vaccinated” if the participant was fully or partially vaccinated or “not vaccinated” if the participant did not receive any dose of the COVID-19 vaccine in the study period. Data on previous infection was also obtained from the serological study with a positive test indicating previous infection and a negative test indicating no previous infections.

### Statistical analysis

All analyses were conducted in R statistical software (v4.0.3, Vienna, Austria). Due to minimal overlap of participants in the HELIUS sub-studies, data analyses were performed across four datasets representing the study populations (as opposed to one fully merged dataset). An overview of the datasets and statistical analyses conducted in each of them is presented in Appendix 1. The first dataset consisted of participants from the *main HELIUS cohort* who were merged with GGD Amsterdam PCR test data. The second dataset consisted of participants from the *COVID-19 serological sub-study* who were merged with GGD Amsterdam PCR test data. The third dataset consisted of participants from the *COVID-19 online sub-study* who were merged with GGD Amsterdam PCR test data. The fourth dataset included all participants from the COVID-19 serological sub-study irrespective of PCR test data merging.

In the first dataset, baseline characteristics were assessed and presented as means (standard deviations; SD) or as proportions by migration background. Age and sex adjusted proportions of SARS-CoV-2 positive PCR tests were calculated via *DirectStandardisation* package, by migration background. Robust Poisson regressions (*via mfx package*) were used to measure associations between SARS-CoV-2 PCR test result (outcome) and migration background (exposure). Prevalence ratios (PRs) and their 95% confidence intervals were calculated. Population attributable fractions (PAFs) were calculated from PRs and population distributions in Amsterdam using the standard formula [17]. PAF were calculated to estimate the fraction (quantify) of all SARS-CoV-2 infections that would not have occurred if there had been no differences by migration background [17]. Initially, age and sex were adjusted for as potential confounders. Subsequently, pre-pandemic sociodemographic and medical factors, as well as pre-pandemic lifestyle factors were alternately adjusted for as explanatory factors (i.e., as covariates in the models and without interaction terms). Relative changes in PAFs (decrease/increase) from age and sex adjusted models to models with pre-pandemic factors were used to assess relative contributions of pre-pandemic factors to SARS-CoV-2 infections risk by migration background. Confidence intervals for the PAFs were not calculated because we used the actual/precise population distribution figures of Amsterdam (as opposed to estimated prevalence which bear some uncertainty) [17]. All statistical tests were two-tailed with an alpha of 0.05.

In the second dataset, similar procedures were used. However, intra-pandemic COVID-19 risk aggravating activities were controlled for in the models rather than pre-pandemic factors. In a similar vein, the third dataset adjusted for intra-pandemic risk mitigating activities rather than pre-pandemic factors. To rule out the possibility that a SARS-CoV-2 PCR test occurred before the initiation of an explanatory activity, only PCR tests performed after the date of study participation was used in both analyses.

### Sensitivity analyses

Sensitivity analyses were performed to determine the consistency of our main findings across the first three datasets and to rule out the possibility of SARS-CoV-2 testing bias. To assess the comparability of the study populations, the distribution of sociodemographic and medical factors was compared across the first three datasets, as these variables were available in all datasets. Additionally, the relative changes in PAFs in the first three datasets were compared to determine if adjustments for sociodemographic and medical factors consistently resulted in larger changes compared to adjusting for other explanatory factors in the base models. To investigate potential SARS-CoV-2 testing bias, the proportions of SARS-CoV-2 antibody testing (indicating past infection) versus PCR testing (indicating current infection) in the second dataset were compared by migration background. Furthermore, to eliminate the possibility of SARS-CoV-2 testing bias influencing the main findings, the main analyses based on SARS-CoV-2 PCR test data were replicated in the fourth dataset using SARS-CoV-2 antibody tests. This involved analysing data from all participants in the COVID-19 serological sub-study, irrespective of data merging.

Additional sensitivity analyses were also conducted to confirm that the vaccination status against COVID-19 and previous COVID-19 infections did not impact our findings on intra-pandemic factors. These analyses were performed using the second time point of the serological study, during which vaccination was widely accessible to the public, and serological data on previous infections was available. To achieve this, we re-run the analysis on intra-pandemic risk aggravating factors while excluding participants who had received vaccinations or had previous infections.

## Results

### Baseline characteristics

**Fig. 1:**
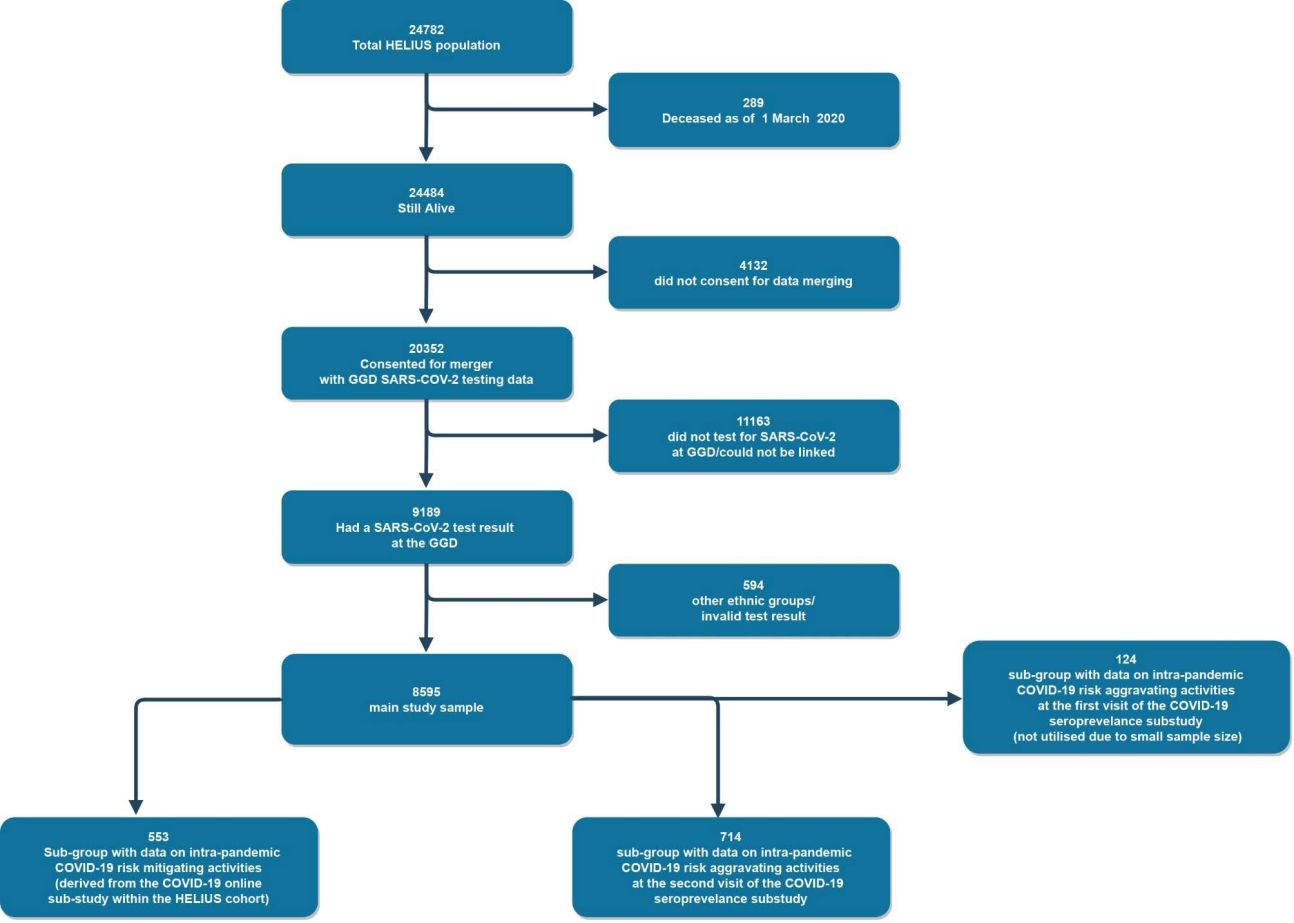
Flow chart of participation. Chart depicts how the final study sample was arrived at

From 20,352 HELIUS participants who gave permission for data linkage and were alive on March 1, 2020, a total of 8,595 were linked to GGD Amsterdam PCR test data and included in the study (Fig. 1). Most participants (40.3%) were of Dutch origin and the least group of participants was from Ghanaian origin (5.8%). Majority of participants were female (58.7%) and had a mean age of 50 years (SD = 13 years). Majority of participants had adequate healthy literacy (87.9%). Populations with a migration background had mostly migrated themselves (77. 1%) and were likely to have lower education and occupational levels, larger households, underlying health conditions, and less physical activity than the Dutch origin population (Table 1).

**Table 1:** Baseline characteristics of participants

Variable name & categories	Total N = 8595	Dutch origin N = 2038	South Asian Surinamese origin N = 1316	African Surinamese origin N = 1605	Ghanaian origin N = 501	Turkish origin N = 1578	Moroccan origin N = 1557
	** N (%)**	**N (%)**	**N (%)**	**N (%)**	**N (%)**	**N (%)**	**N (%)**
**Sex**							
Females	5048(58.7)	1130(55.4)	750(57.0)	1024(63.8)	305(60.9)	884(56.0)	955(61.3)
Males	3547(41.3)	908(44.6)	566(43.0)	581(36.2)	196(39.1)	694(44.0)	602(38.7)
**Age in years (**as of 1 February 2021**)**							
Mean age (SD)	49.8(13.1)	51.4(13.7)	51.9(13.3)	53.8(12.4)	50.5(11.4)	46.1(11.7)	45.3(12.3)
24–29 years	707(8.2)	114(5.6)	95(7.2)	70(4.4)	38(7.6)	185(11.7)	205(13.2)
30–34 years	758(8.8)	197(9.7)	99(7.5)	96(6.0)	30(6.0)	150(9.5)	186(11.9)
35–39 years	838(9.7)	220(10.8)	109(8.3)	116(7.2)	33(6.6)	173(11.0)	187(12.0)
40–44 years	838(9.7)	189(9.3)	94(7.1)	117(7.3)	44(8.8)	195(12.4)	199(12.8)
45–49 years	939(10.9)	201(9.9)	123(9.3)	137(8.5)	56(11.2)	207(13.1)	215(13.8)
50–54 years	1136(13.2)	221(10.8)	177(13.4)	204(12.7)	85(17.0)	259(16.4)	190(12.2)
55–59 years	1256(14.6)	243(11.9)	214(16.3)	285(17.8)	108(21.6)	244(15.5)	162(10.4)
60–64 years	1022(11.9)	256(12.6)	186(14.1)	283(17.6)	80(16.0)	106(6.7)	111(7.1)
65–69 years	625(7.3)	206(10.1)	123(9.3)	179(11.2)	20(4.0)	35(2.2)	62(4.0)
70–74 years	389(4.5)	159(7.8)	76(5.8)	92(5.7)	7(1.4)	21(1.3)	34(2.2)
75–79 years	87(1.0)	32(1.6)	20(1.5)	26(1.6)	0(0.0)	3(0.2)	6(0.4)
**Education**							
No school/Low education	1216(14.1)	57(2.8)	168(12.8)	67(4.2)	122(24.4)	435(27.6)	367(23.6)
Lower secondary education	2075(24.1)	241(11.8)	443(33.7)	521(32.5)	176(35.1)	405(25.7)	289(18.6)
Upper secondary education	2644(30.8)	439(21.5)	400(30.4)	595(37.1)	163(32.5)	479(30.4)	568(36.5)
Tertiary/higher vocational education	2602(30.3)	1293(63.4)	299(22.7)	409(25.5)	34(6.8)	247(15.7)	320(20.6)
**Occupation**							
Elementary Occupation	890(10.4)	24(1.2)	123(9.3)	98(6.1)	244(48.7)	228(14.4)	173(11.1)
Lower occupation	2163(25.2)	280(13.7)	393(29.9)	469(29.2)	126(25.1)	487(30.9)	408(26.2)
Medium occupation	2057(23.9)	434(21.3)	370(28.1)	538(33.5)	45(9.0)	307(19.5)	363(23.3)
Higher occupation	1625(18.9)	739(36.3)	221(16.8)	304(18.9)	17(3.4)	135(8.6)	209(13.4)
Scientific occupation	629(7.3)	417(20.5)	57(4.3)	52(3.2)	8(1.6)	57(3.6)	38(2.4)
**Migration generation**							
1st generation	4892(56.9)	NA	990(75.2)	1311(81.7)	463(92.4)	1096(69.5)	1032(66.3)
2nd generation	1665(19.4)	NA	326(24.8)	294(18.3)	38(7.6)	482(30.5)	525(33.7)
No migration history	2038(23.7)	2038(100)	NA	NA	NA	NA	NA
**Health literacy**							
Health literacy adequate	7558(87.9)	2024(99.3)	1224(93.0)	1557(97.0)	346(69.1)	1159(73.4)	1248(80.2)
Health literacy not adequate	1005(11.7)	14(0.7)	87(6.6)	45(2.8)	149(29.7)	408(25.9)	302(19.4)
**Household size**							
1 person household	1546(18.0)	520(25.5)	268(20.4)	435(27.1)	67(13.4)	115(7.3)	141(9.1)
2-person household	1998(23.2)	772(37.9)	302(22.9)	403(25.1)	106(21.2)	218(13.8)	197(12.7)
3-person household	1651(19.2)	339(16.6)	293(22.3)	340(21.2)	125(25.0)	318(20.2)	236(15.2)
4-person household	1766(20.5)	318(15.6)	277(21.0)	263(16.4)	112(22.4)	474(30.0)	322(20.7)
5-person household +	1553(18.1)	84(4.1)	166(12.6)	141(8.8)	82(16.4)	437(27.7)	643(41.3)
**Underlying health conditions** ^**1**^							
Underlying health conditions on medications	1003(11.7)	213(10.5)	203(15.4)	245(15.3)	60(12.0)	174(11.0)	108(6.9)
Underlying health conditions not on medications	3700(43.0)	579(28.4)	600(45.6)	824(51.3)	287(57.3)	749(47.5)	661(42.5)
No underlying health conditions	3892(45.3)	1246(61.1)	513(39.0)	536(33.4)	154(30.7)	655(41.5)	788(50.6)
**Physical activity** ^**2**^							
Met recommendations	4884(56.8)	1562(76.6)	693(52.7)	955(59.5)	291(58.1)	640(40.6)	743(47.7)
Did not meet recommendation	3701(43.1)	474(23.3)	621(47.2)	650(40.5)	210(41.9)	935(59.3)	811(52.1)
**Smoking**							
Current	2022(23.5)	503(24.7)	354(26.9)	456(28.4)	21(4.2)	502(31.8)	186(11.9)
Never	4700(54.7)	747(36.7)	763(58.0)	846(52.7)	424(84.6)	749(47.5)	1171(75.2)
Past	1840(21.4)	783(38.4)	191(14.5)	300(18.7)	54(10.8)	317(20.1)	195(12.5)
**Alcohol consumption**							
Yes	4464(51.9)	1875(92.0)	743(56.5)	1108(69.0)	277(55.3)	351(22.2)	110(7.1)
No	4095(47.6)	162(7.9)	566(43.0)	489(30.5)	221(44.1)	1216(77.1)	1441(92.5)
**Fruit intake per week**							
mean (SD)	7.29(4.7)	7.64(4.7)	7.32(4.8)	7.28(4.8)	6.17(4.6)	7.43(4.7)	7.04(4.7)
**SARS-Cov-2 PCR test result**							
COVID-19 PCR positive	2603(30.3)	307(15.1)	388(29.5)	483(30.1)	140(27.9)	618(39.2)	667(42.8)
COVID-19 PCR negative	5992(69.7)	1731(84.9)	928(70.5)	1122(69.9)	361(72.1)	960(60.8)	890(57.2)
**COVID-19 waves**							
Pre-second wave (June - September 2020)	858(10.0)	165(8.1)	123(9.3)	163(10.2)	35(7.0)	151(9.6)	221(14.2)
Second wave (September 2020 -June 2021)	5855(68.1)	1480(72.6)	916(69.6)	997(62.1)	358(71.5)	1111(70.4)	993(63.8)
Post-second wave (June - September 2021)	1841(21.4)	387(19.0)	269(20.4)	438(27.3)	103(20.6)	306(19.4)	338(21.7)

^1^ Meeting the PA goal was defined as at least 30 min/day of moderate- to high- intensity PA 5 days/week

^2^ Underlying health conditions includes one or more of hypertension, diabetes, obesity, kidney disease, asthma, and metabolic syndrome which were available for use in our study

### Intra-pandemic activities

Data on intra-pandemic activities were obtained from the HELIUS serological sub-study, which included two visits (the first in June and 2020, and the second in November and June 2021). Due to limited COVID-19 testing at the start of the pandemic, only 124 sub-study participants were linked to the GGD Amsterdam PCR test data during the first visit (Fig. 1). However, 714 sub-study participants were linked to the GGD Amsterdam PCR test data during the second visit due to widespread COVID-19 testing by this time (Fig. 1). Data from the first sub-study visit were excluded due to small sample size (Fig. 1). From the 714 included participants, those of Dutch origin were more likely to engage in intra-pandemic COVID-19 risk aggravating activities than populations with a migration background (Table 2). During this study period (when data on intra-pandemic activities was collected), Dutch and South-Asian Surinamese groups had higher vaccination proportions against COVID-19 compared to other groups (34.5% and 35.8% respectively) (Table 2). In contrast, Ghanaians were most likely to have had previous infections of COVID-19 in this study period (34.2%) (Table 2). A separate group of 553 participants provided responses on intra-pandemic COVID-19 risk mitigation activities (Fig. 1). According to the responses, intra-pandemic COVID-19 risk-mitigation activities were more commonly performed by the populations with a migration background when compared to the Dutch population (Table 3).

**Table 2:** Distribution of intra-pandemic COVID-19 risk aggravating activities, vaccination status against COVID-19 and previous COVID-19 infections

Variable name & Categories	Total N = 714	Dutch origin N = 206	South Asian Surinamese origin N = 151	African Surinamese origin N = 119	Ghanaian origin N = 38	Turkish origin N = 102	Moroccan origin N = 98
	N (%)	N (%)	N (%)	N (%)	N (%)	N (%)	N (%)
**Went for grocery shopping during coronavirus pandemic**							
Yes	650(91.0)	195(94.7)	128(84.8)	112(94.1)	30(78.9)	91(89.2)	94(92.5)
No	63(8.8)	11(5.3)	22(14.6)	7(5.9)	8(21.1)	11(10.8)	4(7.5)
**Visited family or friends during coronavirus pandemic**							
Yes	356(49.9)	134(65.0)	57(37.7)	49(41.2)	8(21.1)	53(52.0)	55(56.1)
No	357(50.0)	72(35.0)	93(61.6)	70(58.8)	30(78.9)	49(48.0)	43(43.9)
**Walked the dog or to played outside with your children during coronavirus pandemic**							
Yes	116(16.2)	52(25.2)	8(5.3)	7(5.9)	3(7.9)	23(22.5)	23(23.5)
No	597(83.6)	154(74.8)	142(94.0)	112(94.1)	35(92.1)	79(77.5)	75(76.5)
**Went outside to get some fresh air or exercise during coronavirus pandemic**							
Yes	455(63.7)	159(77.2)	87(57.6)	64(53.8)	19(50.0)	68(66.7)	58(59.2)
No	258(36.1)	47(22.8)	63(41.7)	55(46.2)	19(50.0)	34(33.3)	40(40.8)
**Went outside to take care of someone, such as informal care or shopping during coronavirus pandemic**							
Yes	102(14.3)	37(18.0)	17(11.3)	17(14.3)	0(0.0)	12(11.8)	19(19.4)
No	611(85.6)	169(82.0)	133(88.1)	102(85.7)	38(100.0)	90(88.2)	79(80.6)
**Went to pick up your medication or to visit the doctor during coronavirus pandemic**							
Yes	168(23.5)	35(17.0)	37(24.5)	31(26.1)	12(31.6)	34(33.3)	19(19.4)
No	545(76.3)	171(83.0)	113(74.8)	88(73.9)	26(68.4)	68(66.7)	79(80.6)
**Visited the church/mosque/place of worship during coronavirus pandemic**							
Yes	55(7.7)	3(1.5)	10(6.6)	9(7.6)	11(28.9)	13(12.7)	9(9.2)
No	658(92.2)	203(98.5)	140(92.7)	110(92.4)	27(71.1)	89(87.3)	89(90.8)
**Visited the cinema, theatre, concert, or museum during coronavirus pandemic**							
Yes	16(2.2)	13(6.3)	2(1.3)	1(0.8)	0(0.0)	0(0.0)	0(0.0)
No	697(97.6)	193(93.7)	148(98.0)	118(99.2)	38(100.0)	102(100.0)	98(100.0)
**Visited a catering facility (bar/restaurant) during coronavirus pandemic**							
Yes	9(1.3)	5(2.4)	1(0.7)	1(0.8)	0(0.0)	2(2.0)	0(0.0)
No	704(98.6)	201(97.6)	149(98.7)	118(99.2)	38(100.0)	100(98.0)	98(100.0)
**Exercised indoors (e.g., visited a sports club/gym) during coronavirus pandemic**							
Yes	81(11.3)	159(77.2)	87(57.6)	64(53.8)	19(50.0)	68(66.7)	58(59.2)
No	632(88.5)	47(22.8)	63(41.7)	55(46.2)	19(50.0)	34(33.3)	40(40.8)
**Visited a recreational area (e.g., forest, beach, or campsite) during coronavirus pandemic**							
Yes	117(16.4)	66(32.0)	19(12.6)	5(4.2)	2(5.3)	9(8.8)	16(16.3)
No	596(83.5)	140(68.0)	131(86.8)	114(95.8)	36(94.7)	93(91.2)	82(83.7)
**Went outside for any other reason during coronavirus pandemic**							
Yes	168(23.5)	59(28.6)	37(24.5)	25(21.0)	1(2.6)	21(20.6)	25(25.5)
No	545(76.3)	147(71.4)	113(74.8)	94(79.0)	37(97.4)	81(79.4)	73(74.5)
**Used public transport during coronavirus pandemic**							
Yes	222(31.1)	58(28.2)	39(25.8)	53(44.5)	13(34.2)	22(21.6)	37(37.8)
No	491(68.8)	148(71.8)	111(73.5)	66(55.5)	25(65.8)	80(78.4)	61(62.2)
**Received visitors at home during coronavirus pandemic**							
Yes	377(52.8)	133(64.6)	73(48.3)	59(49.6)	9(23.7)	56(54.9)	47(48.0)
No	336(47.1)	73(35.4)	77(51.0)	60(50.4)	29(76.3)	46(45.1)	51(52.0)
**Went to work during coronavirus Pandemic**							
Yes	364(51.0)	100(48.5)	75(49.7)	59(49.6)	19(50.0)	54(52.9)	57(58.2)
No	350(49.0)	106(51.5)	76(50.3)	60(50.4)	19(50.0)	48(47.1)	41(41.8)
**Total number of daily activities during coronavirus pandemic**							
mean (SD)	5.6(1.7)	6.3(1.6)	5.0(1.8)	5.2(1.7)	4.4(1.6)	5.5(1.6)	5.8(1.6)
**Vaccinated against COVID-19**							
Yes	212(29.7)	71(34.5)	54(35.8)	30(25.4)	7(18.4)	28(27.5)	22(22.4)
No	511(70.3)	135(65.5)	97(64.2)	88(74.6)	31(81.6)	74(72.5)	76(77.6)
**Previous COVID-19 infection**							
Yes	53(7.45)	14(6.8)	8(5.3)	5(4.2)	13(34.2)	9(8.8)	4(4.1)
No	658(92.5)	191(93.2)	142(94.7)	113(95.8)	25(65.8)	93(0.912)	94(95.9)

COVID-19 risk aggravating activities were only available in the HELIUS COVID-19 serological sub-study. The HELIUS COVID-19 serological sub-study data used here was collected from November 23, 2020, to March 31, 2021 (during the second wave of coronavirus pandemic in the Netherlands)

Columns do not add to the total number of participants in some cases due to NAs (missing values)

Vaccination against COVID-19 = “vaccinated” if the participant was fully or partially vaccinated or “not vaccinated” if the participant did not receive any dose of the COVID-19 vaccine in the study period

Previous COVID-19 infection = testing positive for SARS-CoV-2 antibodies in the serological study

**Table 3:** Distribution of intrapandemic-COVID-19 risk mitigating activities COVID-19 risk mitigating activities were only available in the HELIUS COVID-19 online sub-study.

Variable name & Categories	Total N = 553	Dutch origin N = 253	South Asian Surinamese origin N = 73	African Surinamese origin N = 95	Ghanaian origin N = 12	Turkish origin N = 60	Moroccan origin N = 60
	N (%)	N (%)	N (%)	N (%)	N (%)	N (%)	N (%)
**How difficult or easy is it for you to regularly wash your hands for 20 s with soap and water?**							
Very easy + easy	425(76.9)	190(75.1)	56(76.7)	78(82.1)	9(75.0)	49(81.7)	43(71.7)
Very difficult + difficult + neutral	107(19.3)	60(23.7)	15(20.5)	14(14.7)	1(8.3)	7(11.7)	10(16.7)
**How difficult or easy is it for you to always cough or sneeze into your elbow (instead of into your hand or in the air)?**							
Very easy + easy	444(80.3)	214(84.6)	51(69.9)	78(82.1)	8(66.7)	51(85.0)	42(70.0)
Very difficult + difficult + neutral	88(15.9)	36(14.2)	20(27.4)	14(14.7)	2(16.7)	5(8.3)	11(18.3)
**How difficult or easy is it for you to always use a paper tissue to wipe or blow your nose (instead of your sleeve or your hand, or a cotton handkerchief)?**							
Very easy + easy	448(81.0)	194(76.7)	60(82.2)	87(91.6)	8(66.7)	52(86.7)	47(78.3)
Very difficult + difficult + neutral	84(15.2)	56(22.1)	11(15.1)	5(5.3)	2(16.7)	4(6.7)	6(10.0)
**How difficult or easy is it for you to stay at home as much as possible?**							
Very easy + easy	220(39.8)	91(36.0)	33(45.2)	50(52.6)	4(33.3)	23(38.3)	19(31.7)
Very difficult + difficult + neutral	312(56.4)	159(62.8)	38(52.1)	42(44.2)	6(50.0)	33(55.0)	34(56.7)
**How difficult or easy is it for you to always stay 1.5 m away from other people (except within your family/household)?**							
Very easy + easy	207(37.4)	79(31.2)	31(42.5)	48(50.5)	6(50.0)	23(38.3)	20(33.3)
Very difficult + difficult + neutral	325(58.8)	171(67.6)	40(54.8)	44(46.3)	4(33.3)	33(55.0)	33(55.0)
**How difficult or easy is it for you to not visit people whose health is already at risk of COVID-19?**							
Very easy + easy	331(59.9)	133(52.6)	46(63.0)	75(78.9)	7(58.3)	40(66.7)	30(50.0)
Very difficult + difficult + neutral	201(36.3)	117(46.2)	25(34.2)	17(17.9)	3(25.0)	16(26.7)	23(38.3)
**How difficult or easy is it for you to not shake hands?**							
Very easy + easy	460(83.2)	219(86.6)	57(78.1)	79(83.2)	7(58.3)	50(83.3)	48(80.0)
Very difficult + difficult + neutral	72(13.0)	31(12.3)	14(19.2)	13(13.7)	3(25.0)	6(10.0)	5(8.3)
**How difficult or easy is it for you to wear a face mask in public?**							
Very easy + easy	361(65.3)	166(65.6)	47(64.4)	70(73.7)	8(66.7)	43(71.7)	27(45.0)
Very difficult + difficult + neutral	171(30.9)	84(33.2)	24(32.9)	22(23.2)	2(16.7)	13(21.7)	26(43.3)

The HELIUS COVID-19 online sub-study data was collected from 27 August 2020 and 29 September 2020 (after the second wave of coronavirus pandemic in the Netherlands)

### Age and sex adjusted proportions of SARS-CoV-2 infections

**Fig. 2:**
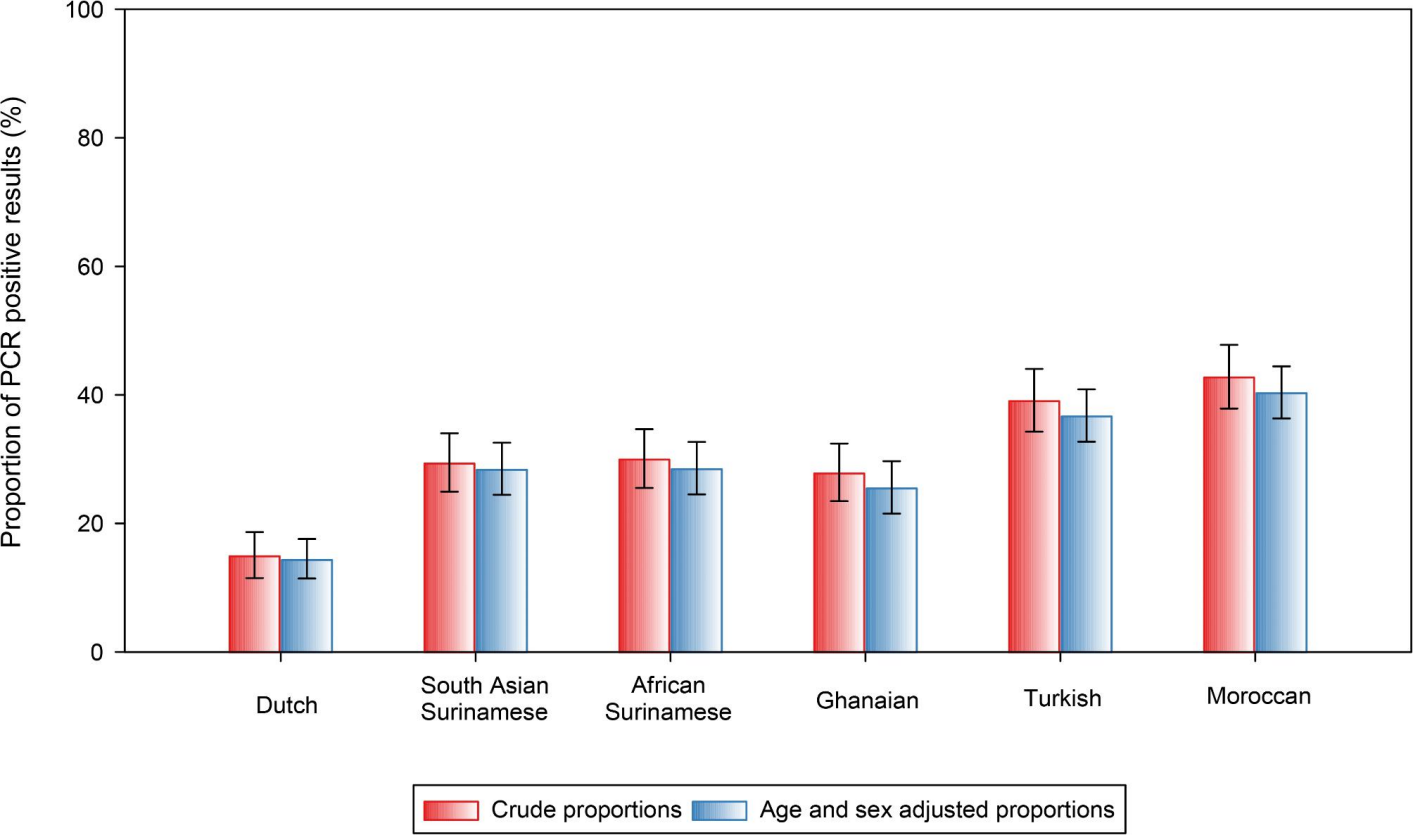
Proportions of PCR positive test results. Bar graph depicts the crude and age and sex adjusted proportions of PCR positive test results by migration background

Out of the 8,595 participants that were linked to GGD Amsterdam PCR test data and included in the study, 2,603 (30.3%) had at least one positive SARS-CoV-2 PCR test (Table 1). Among these participants, age and sex adjusted proportions of SARS-CoV-2 positive PCR tests were higher in populations with a migration background than the Dutch origin population (Fig. 2). In fact, the highest proportions of age and sex adjusted SARS-CoV-2 positive test results were in Turkish and Moroccan origin population (36.8% and 40.4% respectively), and lowest in Dutch origin population (14.5%).

### Relative contributions of pre-pandemic factors and intra-pandemic activities to SARS-CoV-2 infections between migrants and non-migrants

**Fig. 3:**
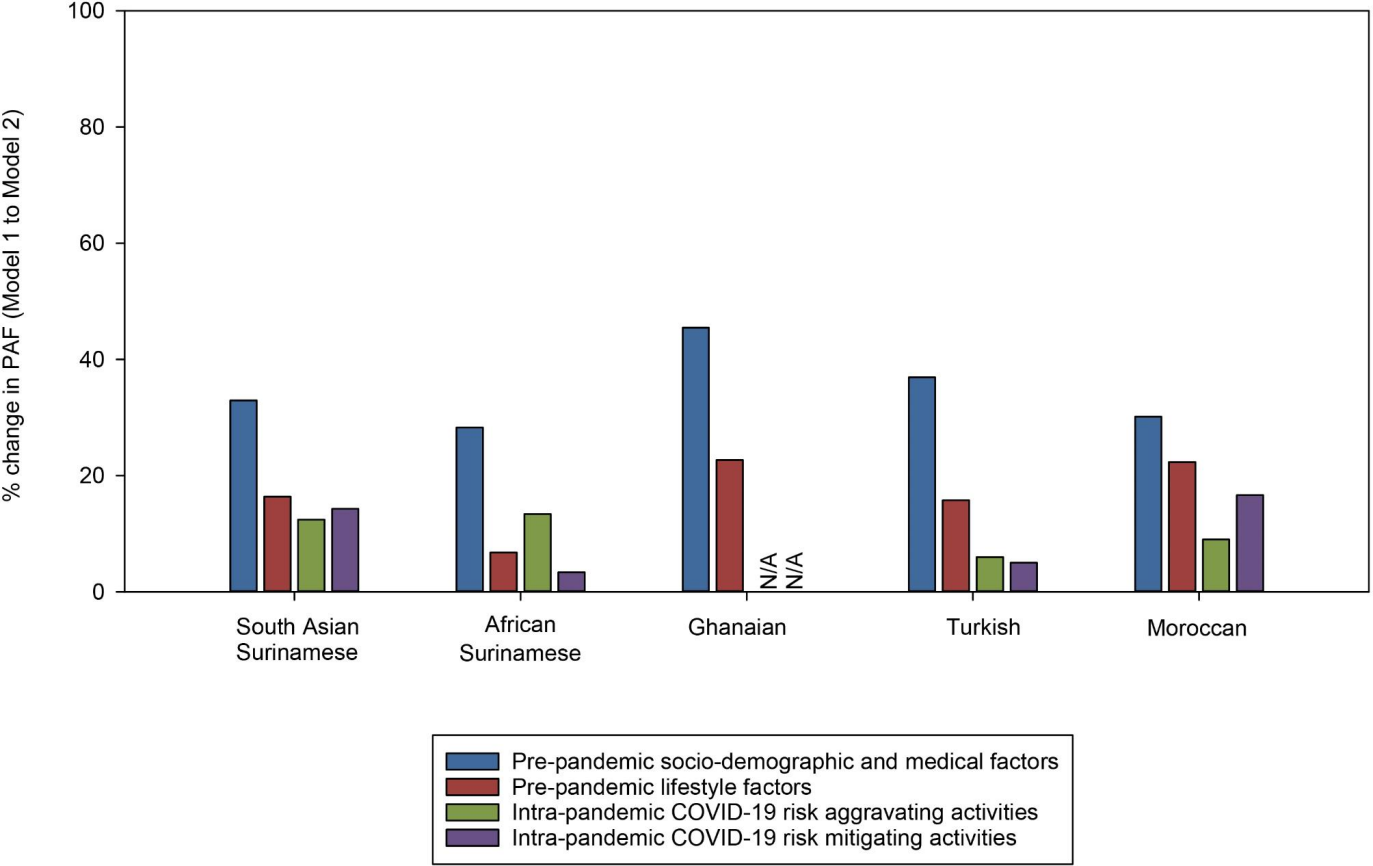
Changes in population attributable fractions. Bar graph depicts percentage change in population attributable fractions from Model 1 to Model 2 by migration background

Age and sex adjusted Poisson regression models across all three study datasets confirmed the higher risk of a PCR positive test result among populations with a migration background than Dutch origin population. The highest risk was in Turkish and Moroccan origin populations (Table 4). By comparing relative changes (decrease/increase) in PAFs from age- and sex-adjusted models to models with explanatory factors across datasets, we observed that pre-pandemic socio-demographic and medical factors resulted in the largest changes in PAFs out of all groups of explanatory factors (up to 45%) (Table 4; Fig. 3). These larger changes in PAFs were also individually observed for education, occupation, and household size (Appendix 2). Pre-pandemic lifestyle factors resulted in moderate changes in PAFs when introduced into the age- and sex-adjusted models, as compared to pre-pandemic socio-demographic and medical factors, or intra-pandemic activities (up to 23%) (Table 4; Fig. 3). These moderate changes were mainly observed with alcohol consumption (Appendix 3). Intra-pandemic activities resulted in the least changes in PAFs compared to pre-pandemic factors when included in age and sex adjusted models (up to 16%) (Table 4; Fig. 3, Appendices 4 & 5).

**Table 4:** Associations between migration background and SARS-Cov-2 positive PCR test, adjusted for pre- pandemic factors and intra- pandemic activities.

Variable	Number per group (N)	Actual population prevalence in Amsterdam (%)	Model 1 PR (95% CI)	Model 1 PAF (%)	Model 2 PR (95% CI)	Model 2 PAF (%)
Pre-pandemic socio-demographic and medical factors (explanatory factors)						
Dutch origin	2038	43.83	1.00 (ref)	ref	1.00(ref)	ref
South Asian Surinamese origin	1316	3.65	1.95(1.68–2.27)	3.35	1.63(1.39–1.93)	2.25
African Surinamese origin	1605	3.65	2.00(1.74–2.31)	3.52	1.71(1.46–2.01)	2.53
Ghanaian origin	501	1.49	1.83(1.49–2.23)	1.22	1.45(1.14–1.83)	0.67
Turkish origin	1578	5.08	2.55(2.23–2.93)	7.30	1.95(1.66–2.32)	4.60
Moroccan origin	1557	8.87	2.80(2.45–3.22)	13.77	2.20(1.87–2.59)	9.62
Pre-pandemic lifestyle factors (explanatory factors)						
Dutch origin	2038	43.83	1.00 (ref)	ref	1.00 (ref)	ref
South Asian Surinamese origin	1316	3.65	1.95(1.68–2.27)	3.35	1.79(1.53–2.11)	2.80
African Surinamese origin	1605	3.65	2.00(1.74–2.31)	3.52	1.93(1.66–2.25)	3.28
Ghanaian origin	501	1.49	1.83(1.49–2.23)	1.22	1.64(1.31–2.02)	0.94
Turkish origin	1578	5.08	2.55(2.23–2.93)	7.30	2.29(1.96–2.70)	6.15
Moroccan origin	1557	8.87	2.80(2.45–3.22)	13.77	2.35(1.99–2.78)	10.70
Intra-pandemic COVID-19 risk aggravating activities (explanatory factors)						
Dutch origin	206	43.83	1.00 (ref)	ref	1.00 (ref)	ref
South Asian Surinamese origin	151	3.65	1.24(1.05–1.48)	0.87	1.21(1.06–1.46)	0.76
African Surinamese origin	119	3.65	1.22(1.01–1.46)	0.80	1.19(1.05–1.43)	0.69
Ghanaian origin	38	1.49	0.92(0.66–1.24)	NA	0.91(0.62–1.21)	NA
Turkish origin	102	5.08	1.33(1.10–1.61)	1.65	1.31(1.06–1.61)	1.55
Moroccan origin	98	8.87	1.43(1.18–1.73)	3.68	1.39(1.14–1.69)	3.35
Intra-pandemic COVID-19 risk mitigating activities (explanatory factors)						
Dutch origin	253	43.83	1.00 (ref)	ref	1.00 (ref)	ref
South Asian Surinamese origin	73	3.65	1.14(1.01–1.42)	0.51	1.18(0.94–1.47)	0.65
African Surinamese origin	95	3.65	1.38(1.20–1.89)	1.37	1.39(1.13–1.69)	1.40
Ghanaian origin	12	1.49	Excluded	Excluded	Excluded	Excluded
Turkish origin	60	5.08	1.50(1.20–1.89)	2.48	1.51(1.19–1.91)	2.53
Moroccan origin	60	8.87	1.49(1.18–1.88)	4.17	1.47(1.13–1.86)	4.00

**Actual population prevalence in Amsterdam**: obtained from Statistics Netherlands as of 01 January 2021. **PR =** Prevalence ratio obtained from Poisson regression models with robust standard errors. Outcome variables = proportion with a SARS-CoV-2 positive PCR test (yes or not). **PAF =** Population attributable fraction calculated from actual prevalence in Amsterdam and prevalence ratios. Confidence intervals for the PAFs were not calculated because we used the actual/precise population figures of Amsterdam (as opposed to estimated prevalence which bear some uncertainty) *Mansournia MA, Altman DG. Population attributable fraction. BMJ 2018 Feb 22;360.***NA =** not applicable. Only applied when PAFs were only calculated when PR was greater than one. **Pre-pandemic socio-demographic and medical factors**: Analysis conducted in in the total dataset (n = 8595). The data spanned from May 2020 (commencement of mass testing) to September 2021. The factors included in the models were: education + occupation + household size + healthy literacy + history of underlying health conditions **Pre-pandemic health-related behaviours**: Analysis conducted in in the total dataset (n = 8595). The data spanned from May 2020 (commencement of mass testing) to September 2021. The factors included in the models were: alcohol consumption + tobacco smoking + physical activity + fruit intake. **Intra-pandemic COVID-19 risk aggravating activities**: Analysis conducted in a subgroup of participants who participated in the COVID-19 serological study (n = 714). The HELIUS COVID-19 serological sub-study data used for current analyses was collected from November 23, 2020, to March 31, 2021 (during the second wave of coronavirus pandemic in the Netherlands). The factors included in the models were: Went for grocery shopping + Visited family or friends + Walked the dog or to played outside with your children + Went outside to get some fresh air or exercise + Went outside to take care of someone, such as informal care or shopping + Went to pick up your medication or to visit the doctor + Visited the church/mosque/place of worship + Visited the cinema, theatre, concert or museum + Visited a catering facility (bar/restaurant) + Exercised indoors (e.g. visited a sports club/gym) + Visited a recreational area (e.g. forest, beach or campsite) + Went outside for any other reason + Used public transport + Received visitors at home + Went to work. **Intra-pandemic COVID-19 risk mitigating activities**: Analysis conducted in a subset group of participants who responded to the online questionnaire about performing COVID-19 mitigating activities during the coronavirus pandemic. The HELIUS COVID-19 online sub-study data used for current analyses was collected from 27 August 2020 to 29 September 2020 (after the first wave of coronavirus pandemic in the Netherlands). Ghanaians were excluded due to low numbers. The factors included in the models were easiness to regularly wash your hands for 20 s with soap and water + easiness to always cough or sneeze into your elbow (instead of into your hand or in the air) + easiness to always use a paper tissue to wipe or blow your nose (instead of your sleeve or your hand, or a cotton handkerchief) + easiness to stay at home as much as possible + easiness for you to always stay 1.5 m away from other people (except within your family/household) + easiness for you to not visit people whose health is already at risk + easiness for you to not shake hands + easiness for you to wear a face mask. **Model 1**: adjusted for age + sex. **Model 2**: Model 1 + (pre-pandemic factors or intra-pandemic activities)

### Sensitivity analyses

First, we found that participants’ socio-demographic and medical characteristics across all main analysis datasets were comparable to those of the total HELIUS population (Table 1, Appendices 6 to 8). Second, across all main analyses’ datasets, we found consistently larger changes in PAFs after controlling for socio-demographic and medical factors in age and sex adjusted models than after controlling for other explanatory factors (Table 4, Appendix 9 & 10). Third, we found that participants of Dutch and Ghanaian origin were less likely to have a positive SARS-CoV-2 PCR test than the other study groups, despite evidence of previous infection (i.e., PCR tests performed before the date of the antibody test indicating past infection, Appendix 11). Fourth, we discovered that PAF reductions from age- and sex-adjusted models to models with explanatory factors were agreeable between SARS-CoV-2 PCR and antibody test results (Appendix 2). Finally, even after excluding participants who had received vaccinations against COVID-19 or had previous COVID-19 infections, we still observed larger changes in (PAFs) after adjusting for socio-demographic and medical factors in age and sex-adjusted models than after controlling for intra-pandemic risk aggravating factors (Appendix 13).

## Discussion

### Summary of findings

In our study on relative contributions of pre-pandemic factors and intra-pandemic activities to differential SARS-CoV-2 infections by migration background, we found that pre-pandemic socio-demographic factors (especially education, occupation, and household size) resulted in the largest changes in PAFs when introduced in age and sex adjusted models, followed by pre-pandemic lifestyle factors (especially alcohol consumption). Intra-pandemic activities resulted in the least changes in PAFs when introduced in age and sex adjusted models.

### Discussion of key findings

Before assessing the relative contributions of pre-pandemic factors and intra-pandemic activities to differential infections by migration background, we confirmed that differences in SARS-CoV-2 infection between migrants and non-migrants were present in our study. We discovered that populations with a migration background had a higher rate of positive SARS-CoV-2 PCR tests than the Dutch origin population. SARS-CoV-2 PCR positivity rates were highest in populations of Turkish and Moroccan origin. This finding is consistent with our previous SARS-CoV-2 antibody-based sub-studies, in which we found that migrant populations had disproportionately higher SARS-CoV-2 infection rates than the Dutch origin population [3]. Our findings also agree with those of other high-income countries, where SARS-CoV-2 infections were found to be significantly higher in migrant populations than in non-migrant populations using both PCR and antibody-based assays [2, 4].

Although the factors that may account for the disproportionately high rates of SARS-CoV-2 infection among migrant populations than non-migrant populations have previously been investigated,[3, 4, 6, 9], we need a better understanding of the factors that contribute most to the observed disparities to better prepare for infectious disease pandemics. We discovered that pre-pandemic socio-demographic factors (particularly education, occupation, and household size) and not intra-pandemic factors contributed the most to PCR assay-based differences in SARS-CoV-2 infections by migration background. The main indicators of socioeconomic status are education and occupation (along with income). As such, they have an impact on a variety of downstream factors. For example, socioeconomic status can influence where one lives, one’s lifestyle, and even one’s level of psychosocial stress [18–20]. Previous research has found that populations with a migration background in the Netherlands have lower socioeconomic status than people of Dutch origin [21]. Consequently, all downstream factors such as neighbourhood environment, diet, psychosocial stress, health seeking behaviours are less favourable in these populations than in the Dutch origin population, potentially leading to an increased risk of SARS-CoV-2 infection [22–24]. Furthermore, the nature of one’s occupation will influence whether one works from home or as an essential worker (e.g., health care worker, taxi driver). Because populations with a migration background tend to have lower-paying jobs and work more often as essential workers, it is not surprising that occupation, as a proxy for virus exposure, contributes significantly to differences in SARS-CoV-2 infections between migrants and non-migrants [23, 24]. Furthermore, populations with a migration background tend to live in larger multi-generational households in the Netherlands. This could explain why, across migrant and non-migrant groups, household size is a major contributor to SARS-CoV-2 infection risk [24].

When compared to both pre-pandemic socio-demographic factors and intra-pandemic activities, pre-pandemic lifestyle factors had intermediate contributions to PCR assay-based SARS-CoV-2 infections by migration background. The category’s largest contribution came from alcohol consumption. A positive relationship between alcohol consumption and viral infections has been previously reported [25]. We found in our study that participants with a migrant background consume less alcohol than participants of Dutch origin. This makes it hard to link the greater risk of SARS-CoV-2 infection observed in populations with a migration background to excessive alcohol use. More research is therefore needed to solve this paradox (i.e., less alcohol use but more SARS-CoV-2 infections).

When compared to pre-pandemic socio-demographic factors or pre-pandemic lifestyle factors, intra-pandemic activities contributed the least to PCR assay-based SARS-CoV-2 infections between migrants and non-migrants. Before interpreting the findings, it should be noted that COVID-19 prevention measures and infection risk are dynamic (i.e., change over time). The intra-pandemic COVID-19 risk-mitigation activities were measured between August and September 2020 (at the beginning of the second wave). This time-period is significant because COVID-19 prevention messages were just beginning to be translated into different languages, and uptake of prevention measures was thought to be lower in populations with a migration background [26]. The intra-pandemic COVID-19 risk-aggravating activities, on the other hand, were measured between November 2020 and March 2021 (in the middle of the second wave). During this period, COVID-19 control measures became more stringent, communication on prevention measures had been translated into several languages, and outreach activities were conducted in neighbourhoods with a high migration background population to improve uptake of the prevention measures [27, 28]. Despite the assumption that uptake of COVID-19 prevention measures would be lower in populations with a migration background than in the Dutch origin population at both time-points, we found that the prevalence of intra-pandemic COVID-19 aggravating activities were lower in populations with a migration background than in the Dutch origin population, while intra-pandemic COVID-19 mitigating activities were slightly higher in populations with a migration background than in the Dutch origin population. While the better profile of COVID-19 risk mitigation among populations with a migration reported in our study could easily explain the low contributions of intra-pandemic activities to the observed differences in PCR assay-based SARS-CoV-2 infections between migrants and non-migrants, other factors could also be at work. For example, the fact that the questions on intra-pandemic mitigating activities were mostly indirect (e.g., how easy/difficult is it to wear face masks) rather than direct (e.g., how frequently you wear a mask) may have led to measurement bias.

Our findings have significant implications for public health. It seems that factors such as education level, occupational status, and household size before the pandemic have a more substantial impact on the differential risk of COVID-19 between migrants and non-migrants, compared to the risk mitigation activities implemented during the pandemic. This highlights the importance of addressing socioeconomic disparities, including improving education, occupation, and household size, among migrant populations. By doing so, we can strive to prevent disproportionately higher infection rates among migrant groups in future infectious disease pandemics.

### Strengths and limitations

Our study has several strengths. First, our study includes pre-pandemic data from 2011 to 2015 as well as intra-pandemic data from 2020 to 2021, allowing us to compare explanatory factors before and during the COVID-19 pandemic. Second, our study included both migrant and non-migrant groups, linked to test result data allowing us to assess SARS-CoV-2 infections using PCR assays by migration background. However, our research has limitations. First, we utilized three datasets to complete our main analyses. Additionally, participants in the online sub-study were invited via email, and both the online and serological sub-studies exhibited low response rates (25% and 23% respectively). Taken together, these factors have the potential to introduce selection and non-response biases into our findings. However, our sensitivity analyses showed that participant characteristics in all three datasets were representative for the total HELIUS population, and the larger changes in PAFs after controlling for socio-demographic and medical factors (in age and gender adjusted models) than after controlling for other explanatory factors were consistent across the study datasets. Second, despite evidence of previous infections (positive SARS-CoV-2 antibody tests), Ghanaians and Dutch origin participants were less likely to go for SARS-CoV-2 PCR test (a test for a recent infection) than other study groups. Despite this SARS-CoV-2 testing bias, our sensitivity analysis revealed that the relative contributions of pre-pandemic factors and intra-pandemic activities to SARS-CoV-2 infection were consistent across both PCR and antibody test results. Consequently, SARS-CoV-2 testing bias observed in our study had probably minimal effect on the primary findings of this study. Third, since questionnaires were used to assess intra-pandemic activities, we cannot rule out measurement bias from a desire to provide socially desirable answers. Fourth, our study relied on household size as a measure of living conditions, rather than using square meters per person. By solely considering household size, we were not able to precisely measure the physical space available to each individual within the household. The use of square meters per person would have provided a more precise and comprehensive assessment of the living space and overcrowding, enabling a more accurate analysis of infection risk factors. Fifth, data on individuals who died from COVID-19 or were hospitalised from COVID-19 were not available for our study. The absence of these variables in our study may lead to an incomplete understanding of the impact of risk factors on SARS-CoV-2 infections among migrant and non-migrant groups, as some migrant groups experienced more severe outcomes than the non-migrant groups [29]. As a result, the study’s findings may not fully capture the association between risk factors and severe cases or mortality. Lastly, data on pre-pandemic factors were collected five to ten years before the COVID-19 pandemic, but these factors could have changed by the time the pandemic began. However, our findings are still important as they highlight how the socio-demographic and health status of individuals prior to the pandemic can influence the disparities in SARS-CoV-2 infections between migrants and non-migrants, assuming no further changes occur.

## Conclusion

Our study has shown that education, occupation, and household size before the COVID-19 outbreak forms part of the foundation for differences in infection between migrant and non-migrant populations in the Netherlands, while behavioural activities during the pandemic add to this pre-existing risk. Interventions that improve pre-pandemic socioeconomic status (i.e., education and occupation), reduce overcrowding in homes, as well as other drivers of inequalities in health are urgently needed at present to better prevent infection disparities in future pandemics in the Netherlands. Behavioural interventions could then be an additional means in reducing disparities during the future infectious disease pandemics.

## Electronic supplementary material

Below is the link to the electronic supplementary material.

Supplementary Material 1

## Data Availability

Data used for the study is available upon request.
