# Supplementary material for: Relative contributions of pre-pandemic factors and intra-pandemic activities to differential COVID-19 risk among migrant and non-migrant populations in the Netherlands: lessons for future pandemic preparedness
Source: Int J Equity Health. 2023 Jul 4;22:127. doi: 10.1186/s12939-023-01936-0 (PMC10320885; doi:10.1186/s12939-023-01936-0)
Supplement: Supplementary file 1 — Supplementary Material 1 [file 12939_2023_1936_MOESM1_ESM.pdf]

Appendix 1. Overview of datasets used for study analyses.

Dataset	Participant description	Total Number	Period	Main analyses	Sensitivity analyses
**1**	participants from the ***main HELIUS cohort*** that had successfully merged with GGD Amsterdam PCR test data	8985	2011- 2015	• Tabulation of baseline characteristics<br>• Calculation of age and sex adjusted proportions of SARS-CoV-2 positive PCR tests.<br>• Calculation of prevalence ratios (PRs) via Poisson regression models<br>• Calculation of population attributable fractions (PAFs)<br>• Adjustments for age and sex as baseline models<br>• Adjustments of sociodemographic and medical factors in models<br>• Adjustments of lifestyle factors in models<br>• Reporting relative changes in PAFs after adjustments.	• Comparison of baseline characteristics with total HELIUS population<br>• Comparison of baseline characteristics with analysis other datasets
**2**	participants from the ***COVID-19 serological substudy*** that had successfully merged with GGD Amsterdam PCR test data	714	November 2020 to September 2021	• Tabulation of baseline characteristics<br>• Calculation of prevalence ratios (PRs) via Poisson regression models<br>• Calculation of population attributable fractions (PAFs)<br>• Adjustments for age and sex as baseline models<br>• Adjustments of sociodemographic and medical factors in models<br>• Adjustments of intra-pandemic risk aggravating activities in models<br>• Reporting relative changes in PAFs after adjustments	• Comparison of baseline characteristics with total HELIUS population<br>• Comparison of baseline characteristics with analysis other datasets<br>• Checking that changes in PAFs in this dataset are consistently larger after correcting for sociodemographic and medical factors than after correcting for intra-pandemic COVID-19 aggravating activities in the base models.<br>• Comparing proportions of SARS-CoV-2 antibody testing (past infections) versus PCR testing (current infections)
**3**	participants from the ***COVID-19 online substudy*** that had successfully merged with GGD Amsterdam PCR test data	553	June 2020 to October 2020	• Tabulation of baseline characteristics<br>• Calculation of prevalence ratios (PRs) via Poisson regression models<br>• Calculation of population attributable fractions (PAFs)<br>• Adjustments for age and sex as baseline models<br>• Adjustments of sociodemographic and medical factors in models<br>• Adjustments of intra-pandemic risk mitigating activities in models<br>• Reporting relative changes in PAFs after adjustments	• Comparison of baseline characteristics with total HELIUS population<br>• Comparison of baseline characteristics with analysis other datasets<br>• Checking that changes in PAFs in this dataset are consistently larger after correcting for sociodemographic and medical factors than after correcting for intra-pandemic COVID-19 mitigating activities in the base models.<br>• Checking that changes in PAFs after removing those vaccinated against COVID-19 and those with previous infections.
**4**	participants from the ***COVID-19 serological substudy*** irrespective of PCR test data merging.	2033	November 2020 to September 2021	• None	• main analyses based on SARS-CoV-2 PCR test data were replicated using SARS-CoV-2 antibody tests

Appendix 2. Associations between migration background and SARS-Cov-2 positive PCR test, adjusted for pre-pandemic socio-demographic and medical factors in the main dataset.

Variable	Number per group (N)	Actual population prevalence in Amsterdam (%)	Model 1 PR (95% CI)	Model 1 PAF (%)	Model 2 PR (95% CI)	Model 2 PAF (%)	% Change in PAFs from Model 1 to 2
Education and occupation (explanatory factor)							
Dutch origin	2038	43.83	1.00 (ref)	ref	1.00 (ref)	ref	ref
South Asian Surinamese origin	1316	3.65	1.95(1.68-2.27)	3.35	1.70(1.44-2.01)	2.49	25.67
African Surinamese origin	1605	3.65	2.00(1.74-2.31)	3.52	1.74(1.49-2.04)	2.63	25.28
Ghanaian origin	501	1.49	1.83(1.49-2.23)	1.22	1.52(1.20-1.91)	0.77	36.89
Turkish origin	1578	5.08	2.55(2.23-2.93)	7.30	2.12(1.81-2.51)	5.38	26.30
Moroccan origin	1557	8.87	2.80(2.45-3.22)	13.77	2.40(2.05-2.82)	11.05	19.75
Household size (explanatory factor)							
Dutch origin	2038	43.83	1.00 (ref)	ref	1.00 (ref)	ref	ref
South Asian Surinamese origin	1316	3.65	1.95(1.68-2.27)	3.35	1.85(1.59-2.16)	3.01	10.15
African Surinamese origin	1605	3.65	2.00(1.74-2.31)	3.52	1.95(1.69-2.26)	3.35	4.83
Ghanaian origin	501	1.49	1.83(1.49-2.23)	1.22	1.73(1.41-2.12)	1.07	12.30
Turkish origin	1578	5.08	2.55(2.23-2.93)	7.30	2.30(2.01-2.66)	6.19	15.21
Moroccan origin	1557	8.87	2.80(2.45-3.22)	13.77	2.49(2.16-2.88)	11.68	15.18
Healthy literacy (explanatory factor)							
Dutch origin	2038	43.83	1.00 (ref)	ref	1.00 (ref)	ref	ref
South Asian Surinamese origin	1316	3.65	1.95(1.68-2.27)	3.35	1.94(1.67-2.26)	3.32	0.90
African Surinamese origin	1605	3.65	2.00(1.74-2.31)	3.52	2.01(1.74-2.32)	3.56	1.14
Ghanaian origin	501	1.49	1.83(1.49-2.23)	1.22	1.76(1.43-2.16)	1.12	8.20
Turkish origin	1578	5.08	2.55(2.23-2.93)	7.30	2.48(2.15-2.86)	6.99	4.25
Moroccan origin	1557	8.87	2.80(2.45-3.22)	13.77	2.73(2.37-3.13)	13.31	3.34
History of underlying medical condition (explanatory factor)							
Dutch origin	2038	43.83	1.00 (ref)	ref	1.00 (ref)	ref	ref
South Asian Surinamese origin	1316	3.65	1.95(1.68-2.27)	3.35	1.91(1.66-2.22)	3.21	0.90
African Surinamese origin	1605	3.65	2.00(1.74-2.31)	3.52	1.95(1.68-2.25)	3.35	1.14
Ghanaian origin	501	1.49	1.83(1.49-2.23)	1.22	1.77(1.44-2.16)	1.13	8.20

Turkish origin	1578	5.08	2.55(2.23-2.93)	7.30	2.48(2.15-2.86)	6.99	4.25
Moroccan origin	1557	8.87	2.80(2.45-3.22)	13.77	2.73(2.38-3.14)	13.31	3.34

Actual population prevalence in Amsterdam: obtained from Statistics Netherlands as of 01 January 2021

PR= Prevalence ratio obtained from Poisson regression models with robust errors. Outcome variable = proportion with a SARS-CoV-2 positive PCR test (yes or not)

PAF=population attributable fraction calculated from actual prevalence in Amsterdam and prevalence ratios. Confidence intervals for the PAFs were not calculated because we used the actual/precise population figures of Amsterdam (as opposed to estimated prevalence which bear some uncertainty) *Mansournia MA, Altman DG. Population attributable fraction. BMJ 2018 Feb 22;360.*

Model 1: adjusted for age + sex

Model 2: Model 1 + each of the pre-pandemic social demographic and medical factors (education+ occupation+ household size + health literacy+ history of underlying factors)

Appendix 3. Associations between migration background and SARS-Cov-2 positive PCR test, adjusted for pre-pandemic lifestyle factors.

Variable	Number per group (N)	Actual population prevalence in Amsterdam (%)	Model 1 PR (95% CI)	Model 1 PAF (%)	Model 2 PR (95% CI)	Model 2 PAF (%)	% Change in PAFs from Model 1 to 2
Alcohol intake (explanatory factor)							
Dutch origin	2038	43.83	1.00 (ref)	ref	1.00 (ref)	ref	ref
South Asian Surinamese origin	1316	3.65	1.94(1.70-2.22)	3.32	1.81(1.55-2.11)	2.87	14.33
African Surinamese origin	1605	3.65	1.99(1.75-2.27)	3.49	1.92(1.66-2.23)	3.25	7.67
Ghanaian origin	501	1.49	1.82(1.53-2.17)	1.21	1.69(1.38-2.08)	1.02	16.39
Turkish origin	1578	5.08	2.54(2.23-2.89)	7.25	2.22(1.90-2.60)	5.84	20.00
Moroccan origin	1557	8.87	2.78(2.44-3.16)	13.64	2.37(2.01-2.78)	10.84	21.28
Tobacco smoking (explanatory factor)							
Dutch origin	2038	43.83	1.00 (ref)	ref	1.00 (ref)	ref	ref
South Asian Surinamese origin	1316	3.65	1.94(1.70-2.22)	3.32	1.92(1.65-2.24)	3.25	2.99
African Surinamese origin	1605	3.65	1.99(1.75-2.27)	3.49	1.99(1.72-2.30)	3.49	0.00
Ghanaian origin	501	1.49	1.82(1.53-2.17)	1.21	1.78(1.40-2.10)	1.16	4.13
Turkish origin	1578	5.08	2.54(2.23-2.89)	7.25	2.55(2.22-2.94)	7.30	0.00
Moroccan origin	1557	8.87	2.78(2.44-3.16)	13.64	2.67(2.33-3.08)	12.91	6.25
Physical activity (explanatory factor)							
Dutch origin	2038	43.83	1.00 (ref)	ref	1.00 (ref)	ref	ref
South Asian Surinamese origin	1316	3.65	1.94(1.70-2.22)	3.32	1.91(1.65-2.23)	3.21	4.18
African Surinamese origin	1605	3.65	1.99(1.75-2.27)	3.49	1.98(1.72-2.29)	3.45	1.99
Ghanaian origin	501	1.49	1.82(1.53-2.17)	1.21	1.81(1.48-2.21)	1.19	2.46
Turkish origin	1578	5.08	2.54(2.23-2.89)	7.25	2.51(2.19-2.89)	7.12	2.47
Moroccan origin	1557	8.87	2.78(2.44-3.16)	13.64	2.76(2.40-3.17)	13.51	1.89
Fruit intake (explanatory factor)							
Dutch origin	2038	43.83	1.00 (ref)	ref	1.00 (ref)	ref	ref
South Asian Surinamese origin	1316	3.65	1.94(1.70-2.22)	3.32	1.95(1.62-2.28)	3.35	0.00
African Surinamese origin	1605	3.65	1.99(1.75-2.27)	3.49	2.04(1.76-2.36)	3.66	3.98
Ghanaian origin	501	1.49	1.82(1.53-2.17)	1.21	1.87(1.52-2.29)	1.28	4.92

Turkish origin	1578	5.08	2.54(2.23-2.89)	7.25	2.60(2.26-3.00)	7.52	3.01
Moroccan origin	1557	8.87	2.78(2.44-3.16)	13.64	2.86(2.49-3.29)	14.17	2.90

Actual population prevalence in Amsterdam: obtained from Statistics Netherlands as of 01 January 2021

PR= Prevalence ratio obtained from Poisson regression models with robust errors. Outcome variable = proportion with a SARS-CoV-2 positive PCR test (yes or not)

PAF=population attributable fraction calculated from actual prevalence in Amsterdam and prevalence ratios. Confidence intervals for the PAFs were not calculated because we used the actual/precise population figures of Amsterdam (as opposed to estimated prevalence which bear some uncertainty) *Mansournia MA, Altman DG. Population attributable fraction. BMJ 2018 Feb 22;360.*

Model 1: adjusted for age + sex.

Model 2: Model 1 + explanatory factor (alcohol intake + tobacco smoking + physical activity + fruit intake per week)

Appendix 4. Associations between migration background and SARS-CoV-2 positive PCR test, adjusted for intra-pandemic risk aggravating activities.

Variable	Number per group (N)	Actual population prevalence in Amsterdam (%)	Model 1 PR (95% CI)	Model 1 PAF (%)	Model 2 PR (95% CI)	Model 2 PAF (%)	% Change in PAFs from Model 1 to 2
Went for grocery shopping (explanatory factor)							
Dutch origin	206	43.83	1.00 (ref)	ref	1.00 (ref)	ref	ref
South Asian Surinamese origin	151	3.65	1.24(1.05-1.48)	0.87	1.26(1.05-1.49)	0.94	8.05
African Surinamese origin	119	3.65	1.22(1.01-1.46)	0.80	1.22(1.01-1.46)	0.80	0.00
Ghanaian origin	38	1.49	0.92(0.66-1.24)	NA	0.92(0.66-1.26)	NA	NA
Turkish origin	102	5.08	1.33(1.10-1.61)	1.65	1.34(1.10-1.62)	1.70	3.03
Moroccan origin	98	8.87	1.43(1.18-1.73)	3.68	1.43(1.18-1.72)	3.59	2.45
Visited family or friends (explanatory factor)							
Dutch origin	206	43.83	1.00 (ref)	ref	1.00 (ref)	ref	ref
South Asian Surinamese origin	151	3.65	1.24(1.05-1.48)	0.87	1.23(1.02-1.46)	0.83	4.60
African Surinamese origin	119	3.65	1.22(1.01-1.46)	0.80	1.19(0.98-1.44)	0.74	6.25
Ghanaian origin	38	1.49	0.92(0.66-1.24)	NA	0.88(0.64-1.21)	NA	NA
Turkish origin	102	5.08	1.33(1.10-1.61)	1.65	1.32(1.09-1.60)	1.60	3.03
Moroccan origin	98	8.87	1.43(1.18-1.73)	3.68	1.42(1.17-1.71)	3.59	2.45
Walked the dog or to played outside with your children (explanatory factor)							
Dutch origin	206	43.83	1.00 (ref)	ref	1.00 (ref)	ref	ref
South Asian Surinamese origin	151	3.65	1.24(1.05-1.48)	0.87	1.25(1.05-1.50)	0.90	3.45
African Surinamese origin	119	3.65	1.22(1.01-1.46)	0.80	1.22(1.01-1.47)	0.80	0.00
Ghanaian origin	38	1.49	0.92(0.66-1.24)	NA	0.92(0.67-1.25)	NA	NA
Turkish origin	102	5.08	1.33(1.10-1.61)	1.65	1.33(1.10-1.62)	1.65	0.00
Moroccan origin	98	8.87	1.43(1.18-1.73)	3.68	1.43(1.18-1.73)	3.68	0.00
Went outside to get some fresh air or exercise (explanatory factor)							
Dutch origin	206	43.83	1.00 (ref)	ref	1.00 (ref)	ref	ref
South Asian Surinamese origin	151	3.65	1.24(1.05-1.48)	0.87	1.23(1.03-1.46)	0.83	4.60
African Surinamese origin	119	3.65	1.22(1.01-1.46)	0.80	1.18(0.98-1.44)	0.75	8.75
Ghanaian origin	38	1.49	0.92(0.66-1.24)	NA	0.89(0.64-1.21)	NA	NA

Turkish origin	102	5.08	1.33(1.10-1.61)	1.65	1.32(1.09-1.61)	1.60	3.03
Moroccan origin	98	8.87	1.43(1.18-1.73)	3.68	1.41(1.16-1.70)	3.51	4.62
Went outside to take care of someone, such as informal care or shopping (explanatory factor)							
Dutch origin	206	43.83	1.00 (ref)	ref	1.00 (ref)	ref	ref
South Asian Surinamese origin	151	3.65	1.24(1.05-1.48)	0.87	1.26(1.05-1.49)	0.94	8.05
African Surinamese origin	119	3.65	1.22(1.01-1.46)	0.80	1.22(1.01-1.47)	0.80	0.00
Ghanaian origin	38	1.49	0.92(0.66-1.24)	NA	0.98(0.67-1.25)	NA	NA
Turkish origin	102	5.08	1.33(1.10-1.61)	1.65	1.34(1.11-1.65)	1.70	3.03
Moroccan origin	98	8.87	1.43(1.18-1.73)	3.68	1.42(1.17-1.72)	3.59	2.45
Went to pick up your medication or to visit the doctor (explanatory factor)							
Dutch origin	206	43.83	1.00 (ref)	ref	1.00 (ref)	ref	ref
South Asian Surinamese origin	151	3.65	1.24(1.05-1.48)	0.87	1.25(1.05-1.49)	0.90	3.45
African Surinamese origin	119	3.65	1.22(1.01-1.46)	0.80	1.22(1.01-1.47)	0.80	0.00
Ghanaian origin	38	1.49	0.92(0.66-1.24)	NA	0.92(0.67-1.25)	NA	0.00
Turkish origin	102	5.08	1.33(1.10-1.61)	1.65	1.33(1.10-1.63)	1.65	0.00
Moroccan origin	98	8.87	1.43(1.18-1.73)	3.68	1.43(1.18-1.73)	3.68	0.00
Visited the church/mosque/place of worship (explanatory factor)							
Dutch origin	206	43.83	1.00 (ref)	ref	1.00 (ref)	ref	ref
South Asian Surinamese origin	151	3.65	1.24(1.05-1.48)	0.87	1.24(1.04-1.48)	0.87	0.00
African Surinamese origin	119	3.65	1.22(1.01-1.46)	0.80	1.21(0.99-1.46)	0.76	5.00
Ghanaian origin	38	1.49	0.92(0.66-1.24)	NA	0.89(0.64-1.21)	NA	NA
Turkish origin	102	5.08	1.33(1.10-1.61)	1.65	1.31(1.08-1.60)	1.55	6.06
Moroccan origin	98	8.87	1.43(1.18-1.73)	3.68	1.41(1.17-1.71)	3.51	4.62
Visited the cinema, theatre, concert, or museum (explanatory factor)							
Dutch origin	206	43.83	1.00 (ref)	ref	1.00 (ref)	ref	ref
South Asian Surinamese origin	151	3.65	1.24(1.05-1.48)	0.87	1.24(1.04-1.49)	0.87	0.00
African Surinamese origin	119	3.65	1.22(1.01-1.46)	0.80	1.21(0.99-1.46)	0.76	5.00
Ghanaian origin	38	1.49	0.92(0.66-1.24)	NA	0.91(0.66-1.24)	NA	NA
Turkish origin	102	5.08	1.33(1.10-1.61)	1.65	1.33(1.09-1.61)	1.65	0.00
Moroccan origin	98	8.87	1.43(1.18-1.73)	3.68	1.42(1.17-1.72)	3.59	2.45
Visited a catering facility e.g., bar/restaurant (explanatory factor)							
Dutch origin	206	43.83	1.00 (ref)	ref	1.00 (ref)	ref	ref

South Asian Surinamese origin	151	3.65	1.24(1.05-1.48)	0.87	1.25(1.05-1.49)	0.90	3.45
African Surinamese origin	119	3.65	1.22(1.01-1.46)	0.80	1.21(1.01-1.46)	0.76	5.00
Ghanaian origin	38	1.49	0.92(0.66-1.24)	NA	0.92(0.66-1.24)	NA	NA
Turkish origin	102	5.08	1.33(1.10-1.61)	1.65	1.33(1.10-1.62)	1.65	0.00
Moroccan origin	98	8.87	1.43(1.18-1.73)	3.68	1.42(1.18-1.72)	3.59	2.45
Exercised indoors e.g., visited a sports club/gym (explanatory factor)							
Dutch origin	206	43.83	1.00 (ref)	ref	1.00 (ref)	ref	ref
South Asian Surinamese origin	151	3.65	1.24(1.05-1.48)	0.87	1.26(1.06-1.51)	0.94	8.05
African Surinamese origin	119	3.65	1.22(1.01-1.46)	0.80	1.22(1.01-1.48)	0.80	0.00
Ghanaian origin	38	1.49	0.92(0.66-1.24)	NA	0.93(0.67-1.27)	NA	NA
Turkish origin	102	5.08	1.33(1.10-1.61)	1.65	1.37(1.13-1.67)	1.74	5.45
Moroccan origin	98	8.87	1.43(1.18-1.73)	3.68	1.44(1.19-1.74)	3.76	2.17
Visited a recreational area e.g., forest, beach, or campsite (explanatory factor)							
Dutch origin	206	43.83	1.00 (ref)	ref	1.00 (ref)	ref	ref
South Asian Surinamese origin	151	3.65	1.24(1.05-1.48)	0.87	1.23(1.03-1.47)	0.83	4.60
African Surinamese origin	119	3.65	1.22(1.01-1.46)	0.80	1.18(0.97-1.43)	0.75	6.25
Ghanaian origin	38	1.49	0.92(0.66-1.24)	NA	0.89(0.65-1.21)	NA	NA
Turkish origin	102	5.08	1.33(1.10-1.61)	1.65	1.30(1.06-1.58)	1.50	9.09
Moroccan origin	98	8.87	1.43(1.18-1.73)	3.68	1.40(1.16-1.69)	3.43	6.79
Went outside for any other reason (explanatory factor)							
Dutch origin	206	43.83	1.00 (ref)	ref	1.00 (ref)	ref	ref
South Asian Surinamese origin	151	3.65	1.24(1.05-1.48)	0.87	1.25(1.05-1.49)	0.90	3.45
African Surinamese origin	119	3.65	1.22(1.01-1.46)	0.80	1.22(1.01-1.46)	0.80	0.00
Ghanaian origin	38	1.49	0.92(0.66-1.24)	NA	0.91(0.66-1.25)	NA	NA
Turkish origin	102	5.08	1.33(1.10-1.61)	1.65	1.34(1.10-1.62)	1.70	3.03
Moroccan origin	98	8.87	1.43(1.18-1.73)	3.68	1.43(1.18-1.73)	3.68	0.00
Used public transport (explanatory factor)							
Dutch origin	206	43.83	1.00 (ref)	ref	1.00 (ref)	ref	ref
South Asian Surinamese origin	151	3.65	1.24(1.05-1.48)	0.87	1.25(1.05-1.48)	0.90	3.45
African Surinamese origin	119	3.65	1.22(1.01-1.46)	0.80	1.22(1.01-1.46)	0.80	0.00
Ghanaian origin	38	1.49	0.92(0.66-1.24)	NA	0.92(0.66-1.25)	NA	NA
Turkish origin	102	5.08	1.33(1.10-1.61)	1.65	1.34(1.10-1.62)	1.70	3.03

Moroccan origin	98	8.87	1.43(1.18-1.73)	3.68	1.43(1.18-1.73)	3.68	0.00
Received visitors at home (explanatory factor)							
Dutch origin	206	43.83	1.00 (ref)	ref	1.00 (ref)	ref	ref
South Asian Surinamese origin	151	3.65	1.24(1.05-1.48)	0.87	1.25(1.05-1.48)	0.90	3.45
African Surinamese origin	119	3.65	1.22(1.01-1.46)	0.80	1.23(1.02-1.48)	0.83	0.00
Ghanaian origin	38	1.49	0.92(0.66-1.24)	NA	0.93(0.67-1.26)	NA	NA
Turkish origin	102	5.08	1.33(1.10-1.61)	1.65	1.33(1.09-1.61)	1.70	3.03
Moroccan origin	98	8.87	1.43(1.18-1.73)	3.68	1.44(1.19-1.74)	3.76	0.00
Went to work (explanatory factor)							
Dutch origin	206	43.83	1.00 (ref)	ref	1.00 (ref)	ref	ref
South Asian Surinamese origin	151	3.65	1.24(1.05-1.48)	0.87	1.24(1.05-1.48)	0.87	0.00
African Surinamese origin	119	3.65	1.22(1.01-1.46)	0.80	1.21(1.01-1.47)	0.76	5.00
Ghanaian origin	38	1.49	0.92(0.66-1.24)	NA	0.92(0.66-1.25)	NA	NA
Turkish origin	102	5.08	1.33(1.10-1.61)	1.65	1.34(1.10-1.62)	1.70	3.03
Moroccan origin	98	8.87	1.43(1.18-1.73)	3.68	1.43(1.18-1.73)	3.68	0.00

Analysis conducted in a subgroup of participants who participated in the COVID-19 serological study (n=714). The HELIUS COVID-19 serological sub-study data used for current analyses was collected from November 23, 2020, to March 31, 2021 (during the second wave of coronavirus pandemic in the Netherlands)

Actual population prevalence in Amsterdam: obtained from Statistics Netherlands as of 01 January 2021

PR= Prevalence ratio obtained directly from Poisson regression models with robust standard errors. Outcome variables = proportion with a SARS-CoV-2 positive PCR test (yes or no)

PAF=population attributable fraction calculated from actual prevalence in Amsterdam and prevalence ratios. Confidence intervals for the PAFs were not calculated because we used the actual/precise population figures of Amsterdam (as opposed to estimated prevalence which bear some uncertainty) *Mansournia MA, Altman DG. Population attributable fraction. BMJ 2018 Feb 22;360.*

NA =not applicable. Only applied when PAFs were only calculated when PR was greater than one.

Model 1: adjusted for age + sex.

Model 2: Model 1 + each explanatory factor separately (Went for grocery shopping , or Visited family or friends or Walked the dog, or to played outside with your children, or Went outside to get some fresh air, or exercise or Went outside to take care of someone, such as informal care or shopping, or Went to pick up your medication or to visit the doctor, or Visited the church/mosque/place of worship, or Visited the cinema, theatre, concert or museum or Visited a catering facility (bar/restaurant) or Exercised indoors (e.g. visited a sports club/gym) or Visited a recreational area (e.g. forest, beach or campsite), or Went outside for any other reason or Used public transport ,or Received visitors at home or Went to work).

Appendix 5. Associations between migration background and SARS-CoV-2 positive PCR test, adjusted for mitigating activities during the coronavirus pandemic.

Variable	Number per group (N)	Actual population prevalence in Amsterdam (%)	Model 1 PR (95% CI)	Model 1 PAF (%)	Model 2 PR (95% CI)	Model 2 PAF (%)	% Change in PAFs from Model 1 to 2
Easiness to regularly wash hands for 20 seconds with soap and water (explanatory factor)							
Dutch origin	253	43.83	1.00 (ref)	ref	1.00 (ref)	ref	ref
South Asian Surinamese origin	73	3.65	1.14(1.01-1.42)	0.51	1.15(0.92-1.43)	0.54	5.88
African Surinamese origin	95	3.65	1.38(1.20-1.89)	1.37	1.36(1.12-1.64)	1.30	5.11
Ghanaian origin	12	1.49	Excluded	Excluded	Excluded	Excluded	Excluded
Turkish origin	60	5.08	1.50(1.20-1.89)	2.48	1.49(1.18-1.89)	2.43	2.02
Moroccan origin	60	8.87	1.49(1.18-1.88)	4.17	1.47(1.15-1.86)	4.00	4.08
Easiness to always cough or sneeze into your elbow (explanatory factor)							
Dutch origin	253	43.83	1.00 (ref)	ref	1.00 (ref)	ref	ref
South Asian Surinamese origin	73	3.65	1.14(1.01-1.42)	0.51	1.18(1.12-1.65)	0.55	7.84
African Surinamese origin	95	3.65	1.38(1.20-1.89)	1.37	1.37(1.18-1.88)	1.33	2.92
Ghanaian origin	12	1.49	Excluded	Excluded	Excluded	Excluded	Excluded
Turkish origin	60	5.08	1.50(1.20-1.89)	2.48	1.49(1.18-1.88)	2.43	2.02
Moroccan origin	60	8.87	1.49(1.18-1.88)	4.17	1.49(1.16-1.88)	4.17	0.00
Easiness to always use a paper tissue to wipe or blow your nose (explanatory factor)							
Dutch origin	253	43.83	1.00 (ref)	ref	1.00 (ref)	ref	ref
South Asian Surinamese origin	73	3.65	1.14(1.01-1.42)	0.51	1.15(0.92-1.43)	0.54	5.88
African Surinamese origin	95	3.65	1.38(1.20-1.89)	1.37	1.34(1.11-1.63)	1.25	8.76
Ghanaian origin	12	1.49	Excluded	Excluded	Excluded	Excluded	Excluded
Turkish origin	60	5.08	1.50(1.20-1.89)	2.48	1.48(1.17-1.86)	2.38	4.03
Moroccan origin	60	8.87	1.49(1.18-1.88)	4.17	1.45(1.14-1.85)	3.84	7.91
Easiness to stay at home as much as possible (explanatory factor)							
Dutch origin	253	43.83	1.00 (ref)	ref	1.00 (ref)	ref	ref
South Asian Surinamese origin	73	3.65	1.14(1.01-1.42)	0.51	1.15(0.91-1.42)	0.54	5.88
African Surinamese origin	95	3.65	1.38(1.20-1.89)	1.37	1.35(1.11-1.63)	1.26	8.03
Ghanaian origin	12	1.49	Excluded	Excluded	Excluded	Excluded	Excluded

Turkish origin	60	5.08	1.50(1.20-1.89)	2.48	1.50(1.18-1.89)	2.48	0.00
Moroccan origin	60	8.87	1.49(1.18-1.88)	4.17	1.47(1.15-1.87)	4.00	4.08
Easiness for you to always stay 1.5 meters away from other people (explanatory factor)							
Dutch origin	253	43.83	1.00 (ref)	ref	1.00 (ref)	ref	ref
South Asian Surinamese origin	73	3.65	1.14(1.01-1.42)	0.51	1.15(0.92-1.43)	0.54	5.88
African Surinamese origin	95	3.65	1.38(1.20-1.89)	1.37	1.36(1.11-1.64)	1.30	5.11
Ghanaian origin	12	1.49	Excluded	Excluded	Excluded	Excluded	Excluded
Turkish origin	60	5.08	1.50(1.20-1.89)	2.48	1.50(1.18-1.89)	2.48	0.00
Moroccan origin	60	8.87	1.49(1.18-1.88)	4.17	1.47(1.15-1.87)	4.00	4.08
Easiness for you to not visit people whose health is already at risk (explanatory factor)							
Dutch origin	253	43.83	1.00 (ref)	ref	1.00 (ref)	ref	ref
South Asian Surinamese origin	73	3.65	1.14(1.01-1.42)	0.51	1.16(0.93-1.45)	0.56	9.80
African Surinamese origin	95	3.65	1.38(1.20-1.89)	1.37	1.39(1.14-1.69)	1.40	2.19
Ghanaian origin	12	1.49	Excluded	Excluded	Excluded	Excluded	Excluded
Turkish origin	60	5.08	1.50(1.20-1.89)	2.48	1.52(1.21-1.92)	2.57	3.63
Moroccan origin	60	8.87	1.49(1.18-1.88)	4.17	1.47(1.15-1.87)	4.00	4.08
Easiness for you to not shake hands (explanatory factor)							
Dutch origin	253	43.83	1.00 (ref)	ref	1.00 (ref)	ref	ref
South Asian Surinamese origin	73	3.65	1.14(1.01-1.42)	0.51	1.15(0.92-1.43)	0.54	5.88
African Surinamese origin	95	3.65	1.38(1.20-1.89)	1.37	1.36(1.13-1.64)	1.30	5.11
Ghanaian origin	12	1.49	Excluded	Excluded	Excluded	Excluded	Excluded
Turkish origin	60	5.08	1.50(1.20-1.89)	2.48	1.51(1.19-1.89)	2.53	2.02
Moroccan origin	60	8.87	1.49(1.18-1.88)	4.17	1.47(1.15-1.87)	4.00	4.08
Easiness for you to wear a face mask in public spaces (explanatory factor)							
Dutch origin	253	43.83	1.00 (ref)	ref	1.00 (ref)	ref	ref
South Asian Surinamese origin	73	3.65	1.14(1.01-1.42)	0.51	1.15(0.92-1.43)	0.55	7.84
African Surinamese origin	95	3.65	1.38(1.20-1.89)	1.37	1.36(1.13-1.65)	1.31	4.38
Ghanaian origin	12	1.49	Excluded	Excluded	Excluded	Excluded	Excluded
Turkish origin	60	5.08	1.50(1.20-1.89)	2.48	1.51(1.19-1.90)	2.52	1.61
Moroccan origin	60	8.87	1.49(1.18-1.88)	4.17	1.46(1.15-1.86)	3.93	5.76

Analysis conducted in a subset group of participants who responded to the online questionnaire about performing COVID-19 mitigating activities during

the coronavirus pandemic. The HELIUS COVID-19 online sub-study data used for current analyses was collected from 27 August 2020 to 29 September 20
(After the first wave of coronavirus pandemic in the Netherlands) Ghanaians excluded due to low numbers.
Actual population prevalence in Amsterdam: obtained from Statistics Netherlands as of 01 January 2021.
PR= Prevalence ratio obtained from Poisson regression models with robust standard errors. Outcome variables = proportion with a SARS-CoV-2 positive PCR test (yes or not).
PAF=Population attributable fraction calculated from actual prevalence in Amsterdam and prevalence ratios. Confidence intervals for the PAFs were not calculated because we used the actual/precise population figures of Amsterdam (as opposed to estimated prevalence which bear some uncertainty) *Mansournia MA, Altman DG. Population attributable fraction. BMJ 2018 Feb 22;360.*
Model 1: adjusted for age + sex.
Model 2: Model 1 + each explanatory factor separately (Easiness to regularly wash hands for 20 seconds with soap and water, or Easiness to always cough or sneeze into your elbow (instead of into your hand or in the air) , or Easiness to always use a paper tissue to wipe or blow your nose (instead of your sleeve or your hand, or a cotton handkerchief), or Easiness to stay at home as much as possible, or Easiness for you to always stay 1.5 metres away from other people (except within your family/household) , or Easiness for you to not visit people whose health is already at risk, or Easiness for you to not shake hands, or Easiness for you to wear a face mask in public spaces)

Appendix 6. Baseline characteristics of participants in the total HELIUS population

Variable name	Categories	Total N=21902	%	Dutch origin N=4500	%	South Asian Surinamese origin N=2996	%	African Surinamese origin N=4082	%	Ghanaian origin N=2310	%	Turkish origin N=3585	%	Moroccan origin N=3889	%
**Sex**	Females	12710	58.0	2451	54.5	1658	55.3	2505	61.4	1422	61.6	1973	55.0	2383	61.3
	Males	9192	42.0	2049	45.5	1338	44.7	1577	38.6	888	38.4	1612	45.0	1506	38.7
**Age in years (**as of 1 February 2021**)**	Mean age (SD)	51.2	13.3	52.9	14.1	52.6	13.5	54.9	12.6	52.0	11.3	47.1	12.2	47.0	12.9
	24-29 years	1583	7.2	221	4.9	185	6.2	166	4.1	141	6.1	396	11.0	448	11.5
	30-34 years	1759	8.0	398	8.8	247	8.2	224	5.5	106	4.6	330	9.2	421	10.8
	35-39 years	1959	8.9	469	10.4	247	8.2	271	6.6	120	5.2	381	10.6	439	11.3
	40-44 years	1947	8.9	396	8.8	194	6.5	274	6.7	199	8.6	388	10.8	460	11.8
	45-49 years	2272	10.4	399	8.9	274	9.1	336	8.2	291	12.6	467	13.0	466	12.0
	50-54 years	2767	12.6	450	10.0	392	13.1	466	11.4	366	15.8	548	15.3	484	12.4
	55-59 years	3232	14.8	524	11.6	442	14.8	692	17.0	464	20.1	549	15.3	462	11.9
	60-64 years	2833	12.9	521	11.6	416	13.9	718	17.6	403	17.4	325	9.1	341	8.8
	65-69 years	1979	9.0	509	11.3	333	11.1	558	13.7	176	7.6	119	3.3	225	5.8
	70-74 years	1268	5.8	511	11.4	195	6.5	300	7.3	38	1.6	65	1.8	116	3.0
	75-79 years	303	1.4	102	2.3	71	2.4	77	1.9	6	0.3	17	0.5	27	0.7
**Education**	No school/Low education	3819	17.4	146	3.2	426	14.2	225	5.5	649	28.1	1119	31.2	1197	30.8
	Lower secondary education	5703	26.0	629	14.0	991	33.1	1443	35.4	905	39.2	880	24.5	689	17.7
	Upper secondary education	6376	29.1	981	21.8	878	29.3	1451	35.5	575	24.9	1017	28.4	1290	33.2
	Tertiary/higher vocational education	5803	26.5	2722	60.5	685	22.9	929	22.8	140	6.1	531	14.8	675	17.4
**Occupation**	Elementary Occupation	2918	13.3	76	1.7	283	9.4	261	6.4	1235	53.5	530	14.8	499	12.8
	Lower occupation	5563	25.4	642	14.3	913	30.5	1300	31.8	456	19.7	1098	30.6	975	25.1
	Medium occupation	4933	22.5	982	21.8	828	27.6	1313	32.2	172	7.4	663	18.5	811	20.9
	Higher occupation	3707	16.9	1637	36.4	475	15.9	713	17.5	57	2.5	292	8.1	437	11.2
	Scientific occupation	1374	6.3	893	19.8	136	4.5	106	2.6	20	0.9	109	3.0	87	2.2
**Migration generation**	1st generation	13529	61.8	NA	NA	2284	76.2	3402	83.3	2202	95.3	2518	70.2	2663	68.5
	2nd generation	3873	17.7	NA	NA	712	23.8	680	16.7	108	4.7	1067	29.8	1226	31.5
	No migration history	4500	20.5	4500	100	NA	NA	NA	NA	NA	NA	NA	NA	NA	NA
**Health literacy**	Health literacy adequate	18644	85.1	4467	99.3	2772	92.5	3964	97.1	1490	64.5	2544	71.0	2899	74.5
	Health literacy not adequate	3154	14.4	31	0.7	211	7.0	109	2.7	795	34.4	1016	28.3	965	24.8
**Household size**	1 person household	4473	20.4	1240	27.6	691	23.1	1301	31.9	342	14.8	351	9.8	407	10.5
	2-person household	5268	24.1	1815	40.3	716	23.9	1030	25.2	476	20.6	576	16.1	507	13.0
	3-person household	4055	18.5	680	15.1	641	21.4	783	19.2	537	23.2	730	20.4	593	15.2
	4-person household	4127	18.8	581	12.9	574	19.2	567	13.9	520	22.5	992	27.7	788	20.3
	5-person household +	3687	16.8	166	3.7	340	11.3	328	8.0	370	16.0	896	25.0	1545	39.7

**Underlying health conditions[1]**	Underlying health conditions on medication	2598	11.9	498	11.1	494	16.5	601	14.7	256	11.1	386	10.8	294	7.6
	Underlying health conditions not on medications	9936	45.4	1384	30.8	1376	45.9	2088	51.2	1376	59.6	1735	48.4	1735	44.6
	No underlying health conditions	9368	42.8	2618	58.2	1126	37.6	1393	34.1	678	29.4	1464	40.8	1860	47.8
**Physical activity[2]**	Met recommendations	12508	56.4	3446	75.5	1622	53.3	2532	61.0	1244	53.2	1507	41.7	1823	46.7
	Did not meet recommendation	9626	43.4	1114	24.4	1414	46.5	1615	38.9	1094	46.8	2100	58.1	2076	53.1
**Smoking**	Current	5302	23.9	1129	24.7	861	28.3	1309	31.5	104	4.4	1240	34.3	525	13.4
	Never	12334	55.6	1689	37.0	1757	57.8	2016	48.6	2027	86.7	1700	47.0	2874	73.6
	Past	4421	19.9	1737	38.1	413	13.6	805	19.4	191	8.2	648	17.9	492	12.6
**Alcohol consumption**	Yes	11221	50.6	4151	91.0	1708	56.1	2826	68.1	1101	47.1	813	22.5	286	7.3
	No	10816	48.8	407	8.9	1322	43.5	1294	31.2	1214	51.9	2772	76.7	3603	92.2
**Fruit intake per week**	mean (SD)	6.36	2.4	6.88	2.2	6.38	2.4	6.19	2.5	5.53	2.5	6.47	2.4	6.31	2.42

[1] Meeting the PA goal was defined as at least 30 min/day of moderate- to high- intensity PA 5 days/week

[2] Underlying health conditions includes one or more of hypertension, diabetes, obesity, kidney disease, asthma, and metabolic syndrome which were available for use in our study

Appendix 7. Baseline characteristics of participants in the HELIUS COVID-19 seroprevalence sub-study.

Variable name	Categories	Total[1] N=714	%	Dutch origin N=206	%	South Asian Surinamese origin N=151	%	African Surinamese origin N=119	%	Ghanaian origin N=38	%	Turkish origin N=102	%	Moroccan origin N=98	%
**Sex**	Females	414	58.0	111	53.9	98	64.9	77	64.7	15	39.5	56	54.9	57	58.2
	Males	300	42.0	95	46.1	53	35.1	42	35.3	23	60.5	46	45.1	41	41.8
**Age in years (**as of 1 February 2021**)**	Mean age (SD)	52.5	12.3	53.1	13.8	55.2	10.6	57.5	11.3	54.0	9.9	46.5	10.5	46.3	10.8
	24-29 years	36	5.0	12	5.8	3	2.0	2	1.7	1	2.6	9	8.8	9	9.2
	30-34 years	41	5.7	13	6.3	8	5.3	5	4.2	0	0.0	8	7.8	7	7.1
	35-39 years	58	8.1	25	12.1	6	4.0	3	2.5	3	7.9	8	7.8	13	13.3
	40-44 years	68	9.5	14	6.8	8	5.3	7	5.9	4	10.5	17	16.7	18	18.4
	45-49 years	77	10.8	15	7.3	11	7.3	15	12.6	3	7.9	20	19.6	13	13.3
	50-54 years	99	13.9	23	11.2	30	19.9	7	5.9	6	15.8	20	19.6	13	13.3
	55-59 years	115	16.1	29	14.1	31	20.5	22	18.5	10	26.3	10	9.8	13	13.3
	60-64 years	107	15.0	28	13.6	30	19.9	26	21.8	8	21.1	7	6.9	8	8.2
	65-69 years	69	9.7	26	12.6	16	10.6	19	16.0	3	7.9	1	1.0	4	4.1
	70-74 years	33	4.6	16	7.8	7	4.6	8	6.7	0	0.0	2	2.0	0	0.0
	75-79 years	11	1.5	5	2.4	1	0.7	5	4.2	0	0.0	0	0.0	0	0.0
**Education**	No school/Low education	53	7.4	2	1.0	16	10.6	2	1.7	5	13.2	13	12.7	15	15.3
	Lower secondary education	155	21.7	19	9.2	50	33.1	35	29.4	16	42.1	21	20.6	14	14.3
	Upper secondary education	208	29.1	40	19.4	45	29.8	41	34.5	11	28.9	36	35.3	35	35.7
	Tertiary/higher vocational education	297	41.6	145	70.4	40	26.5	41	34.5	6	15.8	31	30.4	34	34.7
**Occupation**	Elementary Occupation	55	7.7	1	0.5	14	9.3	8	6.7	12	31.6	13	12.7	7	7.1
	Lower occupation	140	19.6	21	10.2	41	27.2	26	21.8	13	34.2	17	16.7	22	22.4
	Medium occupation	178	24.9	41	19.9	43	28.5	40	33.6	3	7.9	27	26.5	24	24.5
	Higher occupation	183	25.6	81	39.3	31	20.5	29	24.4	4	10.5	14	13.7	24	24.5
	Scientific occupation	80	11.2	48	23.3	7	4.6	6	5.0	3	7.9	11	10.8	5	5.1
**Migration generation**	1st generation	410	57.4	NA	NA	128	84.8	103	86.6	37	97.4	72	70.6	70	71.4
	2nd generation	98	13.7	NA	NA	23	15.2	16	13.4	1	2.6	30	29.4	28	28.6
	No migration history	206	28.9	206	100	NA	NA	NA	NA	NA	NA	NA	NA	NA	NA
**Health literacy**	Health literacy adequate	673	94.3	205	99.5	149	98.7	115	96.6	30	78.9	86	84.3	88	89.8
	Health literacy not adequate	39	5.5	1	0.5	2	1.3	4	3.4	8	21.1	14	13.7	10	10.2
**Household size**	1 person household	149	20.9	56	27.2	25	16.6	36	30.3	8	21.1	10	9.8	14	14.3
	2-person household	191	26.8	75	36.4	47	31.1	28	23.5	10	26.3	14	13.7	17	17.3
	3-person household	136	19.0	28	13.6	39	25.8	29	24.4	7	18.4	23	22.5	10	10.2
	4-person household	146	20.4	40	19.4	28	18.5	20	16.8	5	13.2	33	32.4	20	20.4
	5-person household +	89	12.5	7	3.4	12	7.9	5	4.2	7	18.4	21	20.6	37	37.8
**Underlying health conditions[2]**	Underlying health conditions on medication	80	11.2	17	8.3	21	13.9	21	17.6	5	13.2	9	8.8	7	7.1
	Underlying health conditions not on medications	304	42.6	70	34.0	74	49.0	61	51.3	22	57.9	40	39.2	37	37.8
	No underlying health conditions	330	46.2	119	57.8	56	37.1	37	31.1	11	28.9	53	52.0	54	55.1
**SARS-CoV-2 PCR result**	COVID-19 PCR positive	187	26.2	28	13.6	44	29.1	32	26.9	3	7.9	46	45.1	34	34.7
	COVID-19 PCR negative	527	73.8	178	86.4	107	70.9	87	73.1	35	92.1	56	54.9	64	65.3

[1]The HELIUS COVID-19 seroprevalence study used for this study was collected from November 23, 2020, to March 31, 2021 (during the second wave of coronavirus pandemic in the Netherlands)

[2] Underlying health conditions include one or more of hypertension, diabetes, obesity, kidney disease, asthma, and metabolic syndrome which were available for use in our study.

Appendix 8. Baseline characteristics of participants in the HELIUS COVID-19 online sub-study.

Variable name	Categories	Total[1] N=553	%	Dutch origin N=253	%	South Asian Surinamese origin N=73	%	African Surinamese origin N=95	%	Ghanaian origin N=12	%	Turkish origin N=60	%	Moroccan origin N=60	%
**Sex**	Females	318	57.5	153	60.5	45	61.6	62	65.3	5	41.7	30	50.0	23	38.3
	Males	235	42.5	100	39.5	28	38.4	33	34.7	7	58.3	30	50.0	37	61.7
**Age in years (**as of 1 February 2021**)**	Mean age (SD)	52.1	12.7	54.4	13.2	50.7	10.9	57.0	9.5	53.2	10.1	43.5	10.3	44.9	12.4
	24-29 years	25	4.5	10	4.0	2	2.7	0	0.0	0	0.0	8	13.3	5	8.3
	30-34 years	37	6.7	17	6.7	6	8.2	3	3.2	0	0.0	2	3.3	9	15.0
	35-39 years	54	9.8	20	7.9	7	9.6	3	3.2	2	16.7	11	18.3	11	18.3
	40-44 years	60	10.8	20	7.9	9	12.3	6	6.3	2	16.7	14	23.3	9	15.0
	45-49 years	49	8.9	23	9.1	5	6.8	7	7.4	1	8.3	7	11.7	6	10.0
	50-54 years	70	12.7	25	9.9	16	21.9	12	12.6	0	0.0	10	16.7	7	11.7
	55-59 years	83	15.0	35	13.8	12	16.4	25	26.3	3	25.0	3	5.0	5	8.3
	60-64 years	80	14.5	43	17.0	11	15.1	17	17.9	2	16.7	5	8.3	2	3.3
	65-69 years	56	10.1	30	11.9	4	5.5	16	16.8	2	16.7	0	0.0	4	6.7
	70-74 years	35	6.3	27	10.7	1	1.4	6	6.3	0	0.0	0	0.0	1	1.7
	75-79 years	4	0.7	3	1.2	0	0.0	0	0.0	0	0.0	0	0.0	1	1.7
**Education**	No school/Low education	21	3.8	5	2.0	2	2.7	1	1.1	2	16.7	6	10.0	5	8.3
	Lower secondary education	103	18.6	29	11.5	21	28.8	24	25.3	4	33.3	12	20.0	13	21.7
	Upper secondary education	171	30.9	55	21.7	27	37.0	37	38.9	5	41.7	25	41.7	22	36.7
	Tertiary/higher vocational education	258	46.7	164	64.8	23	31.5	33	34.7	1	8.3	17	28.3	20	33.3
**Occupation**	Elementary Occupation	21	3.8	3	1.2	3	4.1	2	2.1	5	41.7	7	11.7	1	1.7
	Lower occupation	105	19.0	30	11.9	15	20.5	24	25.3	4	33.3	16	26.7	16	26.7
	Medium occupation	148	26.8	53	20.9	26	35.6	33	34.7	2	16.7	16	26.7	18	30.0
	Higher occupation	176	31.8	111	43.9	16	21.9	25	26.3	0	0.0	10	16.7	14	23.3
	Scientific occupation	70	12.7	50	19.8	6	8.2	5	5.3	0	0.0	7	11.7	2	3.3
**Migration generation**	1st generation	215	38.9	NA	NA	55	75.3	82	86.3	12	100.0	34	56.7	32	53.3
	2nd generation	85	15.4	NA	NA	18	24.7	13	13.7	0	0.0	26	43.3	28	46.7
	No migration history	253	45.7	253	100	NA	NA	NA	NA	NA	NA	NA	NA	NA	NA
**Health literacy**	Health literacy adequate	538	97.3	253	100.0	72	98.6	93	97.9	9	75.0	56	93.3	55	91.7
	Health literacy not adequate	15	2.7	0	0.0	1	1.4	2	2.1	3	25.0	4	6.7	5	8.3
**Household size**	1 person household	114	20.6	65	25.7	11	15.1	28	29.5	3	25.0	0	0.0	7	11.7
	2-person household	149	26.9	88	34.8	18	24.7	24	25.3	3	25.0	6	10.0	10	16.7
	3-person household	109	19.7	41	16.2	24	32.9	24	25.3	0	0.0	13	21.7	7	11.7
	4-person household	111	20.1	49	19.4	16	21.9	13	13.7	3	25.0	23	38.3	7	11.7
	5-person household +	69	12.5	10	4.0	4	5.5	5	5.3	3	25.0	18	30.0	29	48.3
**Acculturation**	Integrated	253	45.8	NA	NA	60	82.2	91	95.8	12	100.0	44	73.3	46	76.7
	Assimilated	11	2.0	NA	NA	6	8.2	1	1.1	0	0.0	2	3.3	2	3.3
	Separated	28	5.1	NA	NA	6	8.2	3	3.2	0	0.0	12	20.0	7	11.7
	Marginalized	7	1.3	NA	NA	1	1.4	0	0.0	0	0.0	2	3.3	4	6.7
**Underlying health conditions[1]**	Underlying health conditions on medication	68	12.3	25	9.9	13	17.8	21	22.1	2	16.7	4	6.7	3	5.0
	Underlying health conditions not on medications	201	36.3	70	27.7	27	37.0	45	47.4	8	66.7	24	40.0	27	45.0
	No underlying health conditions	284	51.4	158	62.5	33	45.2	29	30.5	2	16.7	32	53.3	30	50.0
**SARS-CoV-2 PCR result**	COVID-19 PCR positive	135	24.4	34	13.4	18	24.7	32	33.7	4	33.3	22	36.7	25	41.7
	COVID-19 PCR negative	418	75.6	219	86.6	55	75.3	63	66.3	8	66.7	38	63.3	35	58.3

[1]The HELIUS COVID-19 online sub-study used for this study was collected from 27 August 2020 and 29 September 2020 (after the second wave of coronavirus pandemic in the Netherlands)

[2]Underlying health conditions includes one or more of hypertension, diabetes, obesity, kidney disease, asthma, and metabolic syndrome which were available for use in our study

Appendix 9. Main analyses on pre-pandemic sociodemographic and medical factors replicated in seroprevalence sub-study: associations between migration background and SARS-CoV-2 PCR test, adjusted for sociodemographic and medical factors.

Variable	Number per group (N)	Actual population prevalence in Amsterdam (%)	Model 1 PR (95% CI)	Model 1 PAF (%)	Model 2 PR (95% CI)	Model 2 PAF (%)	% Change in PAFs from Model 1 to 2
Dutch origin	206	43.83	1.00 (ref)	ref	1.00(ref)	ref	ref
South Asian Surinamese origin	151	3.65	1.24(1.05-1.48)	0.87	1.13(0.92-1.37)	0.47	45.98
African Surinamese origin	119	3.65	1.22(1.01-1.46)	0.80	1.17(0.95-1.44)	0.62	22.50
Ghanaian origin	38	1.49	0.92(0.66-1.24)	NA	0.80(0.56-1.11)	NA	NA
Turkish origin	102	5.08	1.33(1.10-1.61)	1.65	1.13(0.89-1.42)	0.66	60.00
Moroccan origin	98	8.87	1.43(1.18-1.73)	3.68	1.20(0.95-1.51)	1.74	52.72

Poisson regression models performed in the main study group (8595) were repeated in the COVID-19 serological sub-study participants (n=714) to assess the validity of using sub-studies. The HELIUS COVID-19 serological sub-study data used for current analyses was collected from November 23, 2020, to March 31, 2021 (during the second wave of coronavirus pandemic in the Netherlands)

Actual population prevalence in Amsterdam: obtained from Statistics Netherlands as of 01 January 2021

PR= Prevalence ratio obtained from Poisson regression models with robust standard errors. Outcome variables = proportion with a SARS-CoV-2 positive PCR test (yes or not).

PAF=Population attributable fraction calculated from actual prevalence in Amsterdam and prevalence ratios. Confidence intervals for the PAFs were not calculated because we used the actual/precise population figures of Amsterdam (as opposed to estimated prevalence which bear some uncertainty) *Mansournia MA, Altman DG. Population attributable fraction. BMJ 2018 Feb 22;360.*

Model 1: adjusted for age + sex.

Model 2: Model 1 + Pre-pandemic socio-demographic and medical factors (education + occupation + household size + healthy literacy + history of underlying health conditions.)

Appendix 10. Main analyses on pre-pandemic sociodemographic and medical factors replicated in online sub-study: associations between migration background and SARS-CoV-2 positive PCR test, adjusted for pre-pandemic sociodemographic and medical factors.

**Variable**	**Number per group (N)**	**Actual population prevalence in Amsterdam (%)**	**Model 1 PR (95% CI)**	**Model 1 PAF (%)**	**Model 2 PR (95% CI)**	**Model 2 PAF (%)**	**% Change in PAFs from Model 1 to 2**
Dutch origin	253	43.83	1.00 (ref)	ref	1.00(ref)	ref	ref
South Asian Surinamese origin	95	3.65	1.14(1.01-1.42)	0.51	1.17(0.91-1.47)	0.62	17.74
African Surinamese origin	73	3.65	1.38(1.20-1.89)	1.37	1.37(1.11-1.67)	1.33	3.01
Ghanaian origin	12	1.49	Excluded	Excluded	Excluded	Excluded	Excluded
Turkish origin	60	5.08	1.50(1.20-1.89)	2.48	1.61(1.24-2.08)	3.01	17.61
Moroccan origin	60	8.87	1.49(1.18-1.88)	4.17	1.62 (1.23-2.12)	5.21	19.96

Poisson regression models performed in the main study group (8595) were repeated in this COVID-19 online sub-study participants (n=553) to assess validity of using the online sub-study.

Ghanaians excluded due to low numbers. The HELIUS COVID-19 online sub-study data used for current analyses was collected from 27 August 2020 to 29 September 2020 (after the first wave of coronavirus pandemic in the Netherlands

Actual population prevalence in Amsterdam: obtained from Statistics Netherlands as of 01 January 2021

PR= Prevalence ratio obtained from Poisson regression models with robust standard errors. Outcome variables = proportion with a SARS-CoV-2 positive PCR test (yes or not).

PAF=Population attributable fraction calculated from actual prevalence in Amsterdam and prevalence ratios. Confidence intervals for the PAFs were not calculated because we used the actual/precise population figures of Amsterdam (as opposed to estimated prevalence which bear some uncertainty) *Mansournia MA, Altman DG. Population attributable fraction. BMJ 2018 Feb 22;360.*

Model 1: adjusted for age + sex.

Model 2: Model 1 + Pre-pandemic socio-demographic and medical factors (education + occupation + household size + healthy literacy + history of underlying health conditions.)

Appendix 11. Ratio of positive PCR tests to positive antibody COVID-19 tests at the time of antibody testing in the HELIUS COVID-19 serological study.

Ethnicity	N Per group	Positive PCR test	Positive antibody test	Both PCR test and antibody test positive	Crude ratio of positive tests[1]	Age and sex adjusted ratio of positive tests [1]
Total	638	161	222	160	0.73(0.61-0.86)	Not applied
Dutch origin	179	17	33	17	0.52(0.30-0.89)	0.50(0.24-1.06)
South Asian Surinamese origin	115	35	38	34	0.92(0.63-1.36)	0.91(0.60-1.37)
African Surinamese origin	105	26	33	26	0.79(0.51-1.23)	0.81(0.52-1.26)
Ghanaian origin	31	10	17	10	0.59(0.32-1.09)	0.58(0.42-0.81)
Turkish origin	118	44	55	44	0.80(0.58-1.10)	0.80(0.58-1.12)
Moroccan origin	90	29	46	29	0.63(0.43-0.92)	0.63(0.44-0.89)

[1]Ratio is derived as proportion of COVID-19 PCR positive tests divided by proportion of COVID-19 antibody positive tests.
Calculated only for PCR tests that took place before the date of antibody test.
The HELIUS COVID-19 serological sub-study took place between November 23, 2020, and June 4, 2021

Appendix 12. Sensitivity analysis with SARS-CoV-2 antibody tests: associations between migration background and SARS-CoV-2 antibody test, adjusted for pre-pandemic factors or intra-pandemic activities.

Variable	Number per group (N)	Actual population prevalence in Amsterdam (%)	Model 1 PR (95% CI)	Model 1 PAF (%)	Model 2 PR (95% CI)	Model 2 PAF (%)	% Change in PAFs from Model 1 to 2
Pre-pandemic socio-demographic and medical factors (explanatory factors)							
Dutch origin	464	43.83	1.00 (ref)	ref	1.00(ref)	ref	ref
South Asian Surinamese origin	391	3.65	1.60(1.12-2.34)	2.14	1.41(0.94-2.11)	1.44	32.71
African Surinamese origin	361	3.65	1.88(1.32-2.70)	3.11	1.76(1.19-2.62)	2.70	13.18
Ghanaian origin	206	1.49	4.27(3.04-6.09)	4.64	3.07(1.99-4.75)	2.99	35.56
Turkish origin	317	5.08	2.64(1.87-3.78)	7.69	1.99(1.34-3.01)	4.79	37.71
Moroccan origin	294	8.87	2.94(2.09-4.20)	14.69	2.04(1.36-3.09)	8.45	42.48
Pre-pandemic lifestyle factors (explanatory factors)							
Dutch origin	464	43.83	1.00 (ref)	ref	1.00(ref)	ref	ref
South Asian Surinamese origin	391	3.65	1.60(1.12-2.34)	2.14	1.52(1.05-2.24)	1.86	13.08
African Surinamese origin	361	3.65	1.88(1.32-2.70)	3.11	1.83(1.27-2.66)	2.94	5.47
Ghanaian origin	206	1.49	4.27(3.04-6.09)	4.64	4.04(2.81-5.88)	4.33	6.68
Turkish origin	317	5.08	2.64(1.87-3.78)	7.69	2.38(1.63-3.51)	6.55	14.82
Moroccan origin	294	8.87	2.94(2.09-4.20)	14.69	2.54(1.71-3.79)	12.02	18.18
Intra-pandemic COVID-19 risk aggravating activities (explanatory factors)							
Dutch origin	464	43.83	1.00 (ref)	ref	1.00(ref)	ref	ref
South Asian Surinamese origin	391	3.65	1.60(1.12-2.34)	2.14	1.59(1.09-2.32)	2.11	1.40
African Surinamese origin	361	3.65	1.88(1.32-2.70)	3.11	1.83(1.30-2.73)	2.94	5.47
Ghanaian origin	206	1.49	4.27(3.04-6.09)	4.64	3.86(2.55-5.62)	4.20	9.48
Turkish origin	317	5.08	2.64(1.87-3.78)	7.69	2.49(1.74-3.62)	7.04	8.45
Moroccan origin	294	8.87	2.94(2.09-4.20)	14.69	2.86(2.01-4.12)	14.17	3.54

Sensitivity analyses with SARS-CoV-2 antibody tests were performed in the HELIUS COVID-19 serological sub-study. The sub-study data used for current analyses was collected from November 23, 2020, to March 31, 2021 (during the second wave of coronavirus pandemic in the Netherlands)

Actual population prevalence in Amsterdam: obtained from Statistics Netherlands as of 01 January 2021.

PR=Prevalence ratio obtained directly from Poisson regression models with robust standard errors. Outcome variable = proportion with a SARS-CoV-2 positive antibody test (yes or not)

PAF=Population attributable fraction calculated from actual prevalence in Amsterdam and prevalence ratios. Confidence intervals for the PAFs were not calculated because we used the actual/precise population figures of Amsterdam (as opposed to estimated prevalence which bear some uncertainty) *Mansournia MA, Altman DG. Population attributable fraction. BMJ 2018 Feb 22;360.*

Pre-pandemic socio-demographic and medical factors: Analysis conducted in in the total dataset (n=8595). The data spanned from May 2020 (commencement of mass testing) to September 2021. The factors included in the models were: education + occupation + household size + healthy literacy + history of underlying health conditions
Pre-pandemic health-related behaviours: Analysis conducted in in the total dataset (n=8595). The data spanned from May 2020 (commencement of mass testing) to September 2021. The factors included in the models were: alcohol consumption + tobacco smoking + physical activity + fruit intake
Intra-pandemic COVID-19 risk aggravating activities: Analysis conducted in a subgroup of participants who participated in the COVID-19 seroprevalence study (n=714). The HELIUS COVID-19 seroprevalence sub-study data used for current analyses was collected from November 23, 2020, to March 31, 2021 (during the second wave of coronavirus pandemic in the Netherlands). The factors included in the models were: Went for grocery shopping + Visited family or friends + Walked the dog or to played outside with your children + Went outside to get some fresh air or exercise + Went outside to take care of someone, such as informal care or shopping + Went to pick up your medication or to visit the doctor + Visited the church/mosque/place of worship + Visited the cinema, theatre, concert or museum + Visited a catering facility (bar/restaurant) + Exercised indoors (e.g. visited a sports club/gym) + Visited a recreational area (e.g. forest, beach or campsite) + Went outside for any other reason + Used public transport + Received visitors at home + Went to work
Model 1: adjusted for age + sex
Model 2: Model 1 + (pre-pandemic factors or intra-pandemic activities).

Appendix 13. Sensitivity analyses after removing participants who were vaccinated against COVID-19 and those with previous COVID-19 infections in the HELIUS COVID-19 serological sub-study.

Variable	Number per group (N)	Actual population prevalence in Amsterdam (%)	Model 1 PR (95% CI)	Model 1 PAF (%)	Model 2 PR (95% CI)	Model 2 PAF (%)	% Change in PAFs from Model 1 to 2
Pre-pandemic socio-demographic and medical factors (mediating factors)							
Dutch origin	127	43.83	1.00 (ref)	ref	1.00(ref)	ref	ref
South Asian Surinamese origin	93	3.65	1.11(0.89-1.39)	0.39	1.00(0.78-1.28)	0.01	38.03
African Surinamese origin	85	3.65	1.13(0.90-1.42)	0.49	1.06(0.83-1.36)	0.24	25.21
Ghanaian origin	20	1.49	0.92(0.59-1.36)	0.13	0.81(0.49-1.26)	0.29	16.46
Turkish origin	67	5.08	1.29(1.02-1.63)	1.46	1.08(0.81-1.43)	0.40	106.89
Moroccan origin	72	8.87	1.34(1.07-1.67)	2.90	1.05(0.79-1.39)	0.44	246.00
Intra-risk aggravating factors (mediating factors)							
Dutch origin	127	43.83	1.00 (ref)	ref	1.00(ref)	ref	ref
South Asian Surinamese origin	93	3.65	1.11(0.89-1.39)	0.39	1.10(0.87-1.39)	0.36	3.03
African Surinamese origin	85	3.65	1.13(0.90-1.42)	0.49	1.09(0.85-1.38)	0.49	0.00
Ghanaian origin	20	1.49	0.92(0.59-1.36)	0.13	0.91(0.57-1.39)	0.13	0.03
Turkish origin	67	5.08	1.29(1.02-1.63)	1.46	1.28(0.99-1.65)	1.46	0.00
Moroccan origin	72	8.87	1.34(1.07-1.67)	2.90	1.30(1.03-1.65)	2.90	0.00

The HELIUS COVID-19 seroprevalence sub-study data used for current analyses was collected from November 23, 2020, to March 31, 2021 (during the second wave of coronavirus pandemic in the Netherlands, n=464.

Actual population prevalence in Amsterdam: obtained from Statistics Netherlands as of 01 January 2021.

PR=Prevalence ratio obtained directly from Poisson regression models with robust standard errors. Outcome variable = proportion with a SARS-CoV-2 positive antibody test (yes or not)

PAF=Population attributable fraction calculated from actual prevalence in Amsterdam and prevalence ratios. Confidence intervals for the PAFs were not calculated because we used the actual/precise population figures of Amsterdam (as opposed to estimated prevalence which bear some uncertainty) *Mansournia MA, Altman DG. Population attributable fraction. BMJ 2018 Feb 22;360.*

Pre-pandemic socio-demographic and medical factors: Analysis conducted in in the total dataset (n=8595). The data spanned from May 2020 (commencement of mass testing) to September 2021. The factors included in the models were: education + occupation + household size + healthy literacy + history of underlying health conditions.

Pre-pandemic health-related behaviours: Analysis conducted in in the total dataset (n=8595). The data spanned from May 2020 (commencement of mass testing) to September 2021. The factors included in the models were: alcohol consumption + tobacco smoking + physical activity + fruit intake.

Intra-pandemic COVID-19 risk aggravating activities: The factors included in the models were: Went for grocery shopping + Visited family or friends + Walked the dog or to played outside with your children + Went outside to get some fresh air or exercise + Went outside to take care of someone, such as informal care or shopping + Went to pick up your medication or to visit the doctor + Visited the church/mosque/place of worship + Visited the cinema, theatre, concert or museum + Visited a

catering facility (bar/restaurant) + Exercised indoors (e.g. visited a sports club/gym) + Visited a recreational area (e.g. forest, beach or campsite) + Went outside for any other reason + Used public transport + Received visitors at home + Went to work

Model 1: adjusted for age + sex

Model 2: Model 1 + (pre-pandemic factors or intra-pandemic activities).
